# Tel Shiqmona during the Iron Age: A first glimpse into an ancient Mediterranean purple dye ‘factory’

**DOI:** 10.1371/journal.pone.0321082

**Published:** 2025-04-16

**Authors:** Golan Shalvi, Naama Sukenik, Paula Waiman-Barak, Zachary C. Dunseth, Shay Bar, Sonia Pinsky, David Iluz, Zohar Amar, Ayelet Gilboa

**Affiliations:** 1 Institute for the Study of Ancient Culture, University of Chicago, Chicago, Illinois, United States of America; 2 School of Archaeology and Maritime Civilizations, Zinman Institute of Archaeology, University of Haifa, Haifa, Israel; 3 National Treasures Department, Israel Antiquities Authority, Jerusalem, Israel; 4 Sonia & Marco Nadler Institute of Archaeology, Tel Aviv University, Tel Aviv, Israel; 5 Department of Anthropology, University of California San Diego, San Diego, California, United States of America; 6 Recanati Institute for Maritime Studies, University of Haifa, Haifa, Israel; 7 The Mina and Everard Goodman Faculty of Life Sciences, Bar Ilan University, Ramat-Gan, Israel; 8 Environmental Sciences and Agriculture Department, Beit Berl College, Beit Berl, Israel; 9 The Martin (Szusz) Department of Land of Israel Studies and Archaeology, Bar Ilan University, Ramat-Gan, Israel; Austrian Academy of Sciences, AUSTRIA

## Abstract

Purple-dyed textiles, primarily woolen, were much sought after in the Ancient Near East and the Mediterranean, and they adorned the powerful and wealthy. It is commonly assumed that in antiquity, purple dye—extracted from specific species of marine mollusks—was produced in large quantities and in many places around the Mediterranean. But despite numerous archaeological excavations, direct and unequivocal evidence for locales of purple-dye production remains very limited in scope. Here we present Tel Shiqmona, a small archaeological tell on Israel’s Carmel coast. It is the only site in the Near East or around the Mediterranean—indeed, in the entire world—where a sequence of purple-dye workshops has been excavated and which has clear evidence for large-scale, sustained manufacture of purple dye and dyeing in a specialized facility for half a millennium, during the Iron Age (ca. 1100–600 BCE). The number and diversity of artifacts related to purple dye manufacturing are unparalleled. The paper focuses on the various types of evidence related to purple dye production in their environmental and archaeological contexts. We utilize chemical, mineralogical and contextual analyses to connect several categories of finds, providing for the first time direct evidence of the instruments used in the purple-dye production process in the Iron Age Levant. The artifacts from Shiqmona also serve as a first benchmark for future identification of significant purple-dye production sites around the Mediterranean, especially in the Iron Age.

## Introductions

### Structure and aims

We present in this paper Tel Shiqmona (henceforward Shiqmona), a site that can unequivocally be identified as a specialized facility for large-scale and long-term production (~1100–600 BCE) of the lucrative purple dye. This is the first time that unequivocal large-scale production sustained for half a millennium is recognized in the Mediterranean. We first briefly summarize the available direct evidence for purple-dye manufacturing sites around the Mediterranean, focusing on its early stages in the Bronze and Iron Ages. Subsequently, we present the diverse finds from Shiqmona that we consider indicative of purple-dye production. We discuss the chemical analysis employed to identify the dye, and then, based on established archaeological methods and mineralogical analysis, we reconstruct the shape and technology of the specialized clay vats used in the process and their architectural setting. Lastly, we discuss the way(s) in which the archaeological phenomena at Shiqmona may contribute to the ongoing discussion on the definition and identification of purple-production sites around the Mediterranean.

### Purple dye of marine origin

From the 2^nd^ millennium BCE to the fall of Constantinople, textiles dyed in ‘true’/ ‘royal’/ ‘Tyrian’ purple were among the most prestigious commodities around the Mediterranean, worn by royalty, elites, and serving cultic purposes [1–5]. The discovery of the dye and its manufacture process—produced mainly from the hypobranchial gland of three species of Murcidae marine mollusks: *Hexaplex trunculus*, *Bolinus brandaris* and *Stramonita haemastoma* [6]—was the subject of legends already in antiquity (Julius Pollux, *Onomasticon* I, 45–49; see also [4,7–9]). Scholarly literature on the subject is vast; for a recent collection of articles and updated bibliographies, see [10–15]. Several successful attempts to dye fabrics with either natural or synthetic materials have been reported by scholars from around the Mediterranean (e.g., [16,17: Appendix A]), artists [18,19] and religious authorities (e.g., [8]). Some of these attempts followed Pliny’s ancient description of the process, dating to the 1st century CE (*Historia Naturalis* IX, 60; cf. [2,20,21]). These, however, do not necessarily replicate actual ancient processes (certainly not the ones implemented hundreds of years before Pliny), nor do they inform about the installations, artifacts, etc. associated with the production in antiquity.

### Archaeological evidence for East Mediterranean purple-dye production in the Bronze and Iron Ages

The process of dyeing fleeces or fibers with purple dye from marine sources involved several distinct stages, including the harvesting of mollusks, their storage, dye extraction, preparation of the dyeing solution, and the actual dyeing [14]. It is important to distinguish between ‘purple-dye production workshops’, where the primary focus was on the extraction and the production of the dye, and ‘purple dyeing workshops’, where the dye was applied to fibers or fleeces—a differentiation highlighted by Mylona [22]. These stages cannot be presumed a priori to have occurred at the same site [22]. Nonetheless, this distinction is often overlooked in scholarly literature, although see for example [14,23,24].

The evidence for purple-dye production and for dyeing is commonly divided into two categories. Direct evidence comprises purple-stained objects, structures, and sediments (see below and S1 Table). These serve minimally as reliable proof of the occurrence of dye manufacturing at a certain site. Finished products dyed or painted purple, such as textiles, funeral larnakes, or wall paintings, should of course be excluded from this category, since they do not indicate where the dye was produced, nor where fibers, textiles or other objects were dyed.

Indirect evidence for dye production, as defined by most scholars, includes primarily the presence of significant quantities of crushed shells of one or more of the three species of dye-producing mollusks, and various objects and installations that were essential (or believed to be necessary) in the production process. These include, for example, pools, ovens, channels, tools that could have been used to crush the shells and extract the dye, and more (e.g., [22,25–29]). Notably, however, mollusks were also used as food, for construction and for other purposes [16,22,30]. Indirect evidence for the existence of a workshop may prove conclusive when several proxies of production are combined in a proper context (such is the case, for example, in Chryssi on Crete in the first half of the second millennium BCE [26]). Indirect evidence for the dyeing stage is archaeologically very elusive.

Considering the widespread availability of the relevant mollusks around the Mediterranean, the social and economic importance of the purple products, and the visible durability of the dye for millennia (for which see below), direct attestations for its production are surprisingly few. Indirect and thus usually contested evidence of the production of the dye indeed exists in many sites (see references below), but only about 12 sites around the Mediterranean have produced direct, unequivocal evidence for purple-dye manufacturing for over 1400 years (ca. 2000–600 BCE), which we summarize below (see S1 Table).

In the Aegean, the Middle Bronze Age site of Pefka in Crete provides, inter alia, evidence for purple-dyeing, as indicted by dye residue on pottery, identified chemically, but without visible traces [31]. Late Bronze Age Aegina Kolonna in the central Aegean provides direct evidence of a dye-production workshop, including (in addition to a significant number of crushed Muricidae shells) two fragments of a small clay container stained purple, also chemically analyzed, and six other lightly stained potsherds [32]. In Cyprus, Bronze and early Iron Ages sites with direct evidence for at least part of the production process include Pyrgos-Mavroraki (red/purple ‘lumps’/clots) [33]; Hala Sultan Tekke (a mudbrick feature, possibly a basin, preserving traces of purple color, and purple traces in sediments) [34]; and Kition (a large clay vessel stained internally with purple) [35,36]. In the Northern Levant, probable sites include Minet el-Beidha (the harbor of Ugarit on the coast of northern Syria; a stained body sherd of a large clay vessel, not analyzed) [37]; and Sarepta in south Lebanon (six stained body sherds, probably originating from two large containers) [38]. In the Southern Levant, relevant Bronze and Iron Ages sites are Tel Kabri in the ‘Akko Plain (two stained sherds, one of them a rim of a clay vat) [21,39]; nearby Tell Keisan (a pithos stained internally and at least one stained juglet) [40]; Tel ‘Akko (one stained clay vessel) [41]; Tel Dor (two stained sherds, one of them a vat rim, see below); and Shiqmona, on which we elaborate below.

### Tel Shiqmona in brief: Environment and Iron Age occupations, excavations and significance

**Fig 1:**
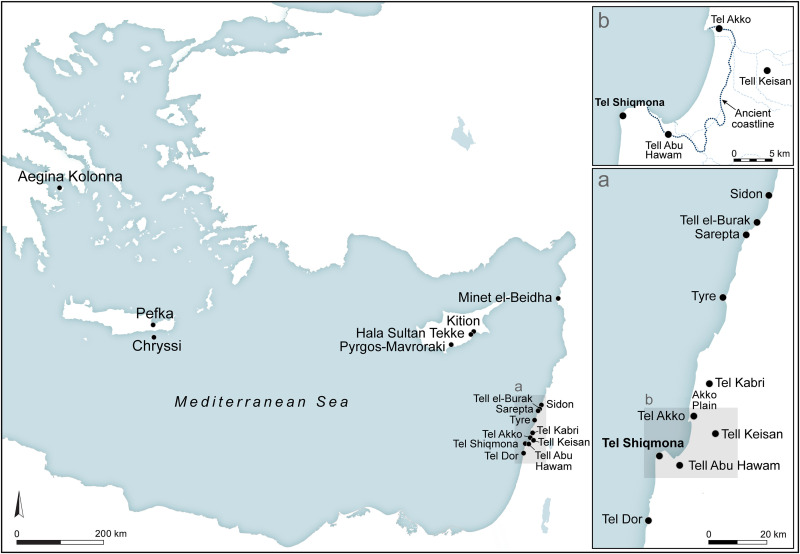
Location map of Tel Shiqmona and other sites mentioned in the text. Ancient coastline in ‘Akko bay following [42]. Map by Sapir Haad.

**Fig 2:**
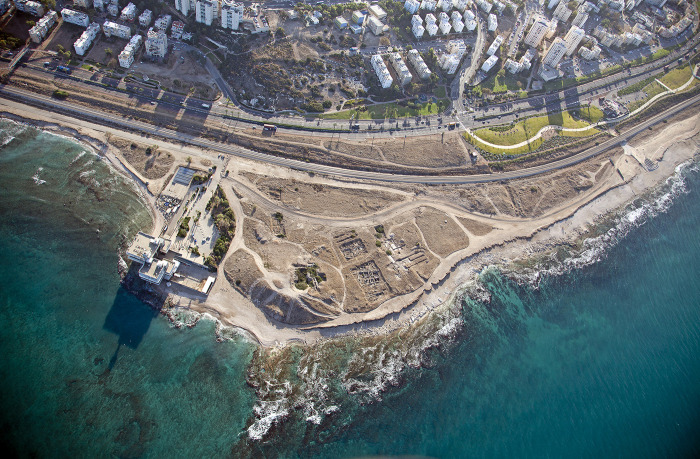
Aerial view of Tel Shiqmona and its surroundings. Looking east. Photo by Michael Eisenberg. Reprinted from [43] under a CC BY license, with permission from the Hecht Museum, University of Haifa, original copyright.

**Fig 3:**
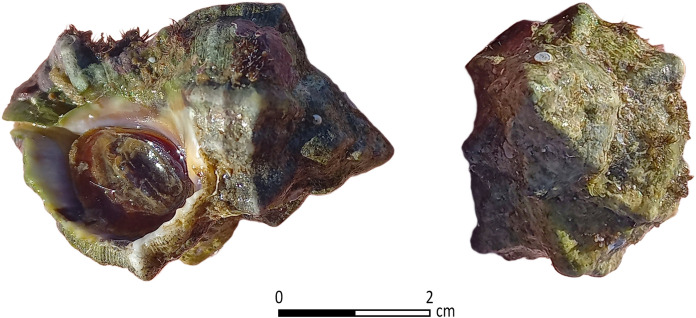
*Hexaplex trunculus* shell collected near Tel Shiqmona. 400 such shells were identified by two free-style divers within 90 mins at a depth of one to two meters on October 20, 2020. Photo by Ayelet Gilboa.

Tel Shiqmona (Tell es-Samak, ‘the mound of fish’ in Arabic) is a small mound of ca. 2 acres, situated on a small rocky headland between the northern tip of the Carmel Mountains and the Mediterranean Sea, today on the southern outskirts of the modern city of Haifa in northern Israel (Figs 1 and 2). During most of the Iron Age, the Carmel coast was a peripheral region between the kingdoms of Israel and Phoenician Tyre. As opposed to contemporaneous coastal sites in the region, Shiqmona has no important anchorage. In fact, because of wind and current regimes, and the wide reef of limestone abrasion platforms alongside the coast (Fig 2), Shiqmona is one of the worst coastal sites for mooring in the Southern Levant [44,45]. On the other hand, these rocky surroundings provide one of the best habitats for marine life in the East Mediterranean littoral, including Muricidae snails, which today has been designated a protected marine park by the Israel Nature and Parks Authority. Mollusk communities in the Eastern Mediterranean basin have been decimated in the last decade to near-extinction by global warming, salinization and pollution [46,47]. However, in the last five years, some recovery has been observed by divers and specialists, particularly around the Shiqmona reef where Muricidae snails are still relatively abundant (Fig 3).

Between 1963 and 1977, Joseph Elgavish conducted extensive excavations at the site on behalf of the Haifa Museums, revealing on the tell a stratigraphic sequence spanning the Late Bronze Age to Byzantine period, and a large Byzantine town flanking the tell (for the Byzantine town and its possible connection to purple dye, see [48]). The Bronze and Iron Ages, however, remained unpublished beyond a short summary [49]. Of the upper/later Iron Age layers, Stratum 7 down to Stratum 13 (see below), Elgavish exposed ca. 800 sq m, which constitutes about 25% of the surface area of the tell. Exposure of the earlier Strata 14 and 15 was much more limited. On a smaller scale, excavations intending to reevaluate the site’s stratigraphy and chronology were renewed by S. Bar and subsequently by G. Shalvi, both of the Zinman Institute of Archaeology at the University of Haifa. The new excavations clarified the stratigraphic sequence of the site, and combined with the study of artifacts from all three excavations, resulted in the definition of ten well-defined and well-dated Iron Age occupations spanning the Iron Age IB to the end of Iron Age IIC, the 11^th^ to late 7^th^ centuries BCE. These dates are based on ceramic chronology in the Southern Levant which, in turn, is supported by radiometric dates from many sites until about 800 BCE (the beginning of the so-called Hallstatt Plateau of the ^14^C calibration curve) and after that are based on correlations between destruction levels at several sites with documented historical events related to the Neo-Assyrian and Neo-Babylonian conquests. For detailed discussions of Shiqmona’s Iron Age chronology, see [50–53]. Shiqmona operated under different local and regional regimes, and it too was repeatedly destroyed and rebuilt (S2 Table).

### Previous studies of evidence for purple dye at Shiqmona

Nira Karmon was the first to study purple dye at Shiqmona [41]. Karmon and Spanier [54,55] defined three categories of evidence:

1) Shell mounds (mostly crushed shells) north and south of the site, which have since been eradicated by modern construction, and thus their exact date and extent is uncertain. The piles comprised shells of the three main species of snails used in purple-dye production and a few other taxa.
2) Numerous Muricidae shells from Elgavish’s excavations found within buildings on the mound.
3) An unspecified number of potsherds stained purple, including vat fragments and rims. Some of these (number not provided) were analyzed by D.H. Abrahams and S. Edelstein and the stains could be verified to originate from marine mollusks [56].

Five more purple-stained vat sherds from Bar’s excavation, dated mainly to the end of the 9^th^ century BCE (four attributed in the current study to Stratum 12 and one to Stratum 13), were analyzed by high-performance liquid chromatography. The analysis proved that the purple was indeed produced from Muricidae sea snails, especially from *Hexaplex trunculus*. Additionally, 1166 Muricidae shells (NISP) from Iron Age levels in Bar’s excavations were classified to the species level, but their exact stratigraphical attribution has not been determined [57].

### Purple-dye production at Iron Age Shiqmona

The profusion of purple-related objects, however, only became clear when—by chance in 2016—we started to identify undocumented purple-stained potsherds in the material excavated by Elgavish. Subsequently, we sorted through all the artifacts saved from Elgavish’s excavations and identified dozens of purple-stained objects both visually and using a Dino-Lite Digital Microscope. With a reevaluation of Elgavish’s excavations and the establishment of a detailed stratigraphy, Shiqmona now affords a high-resolution diachronic perspective of purple dye manufacture over half a millennium.

## Materials and methods

### Artifact database

**Fig 4:**
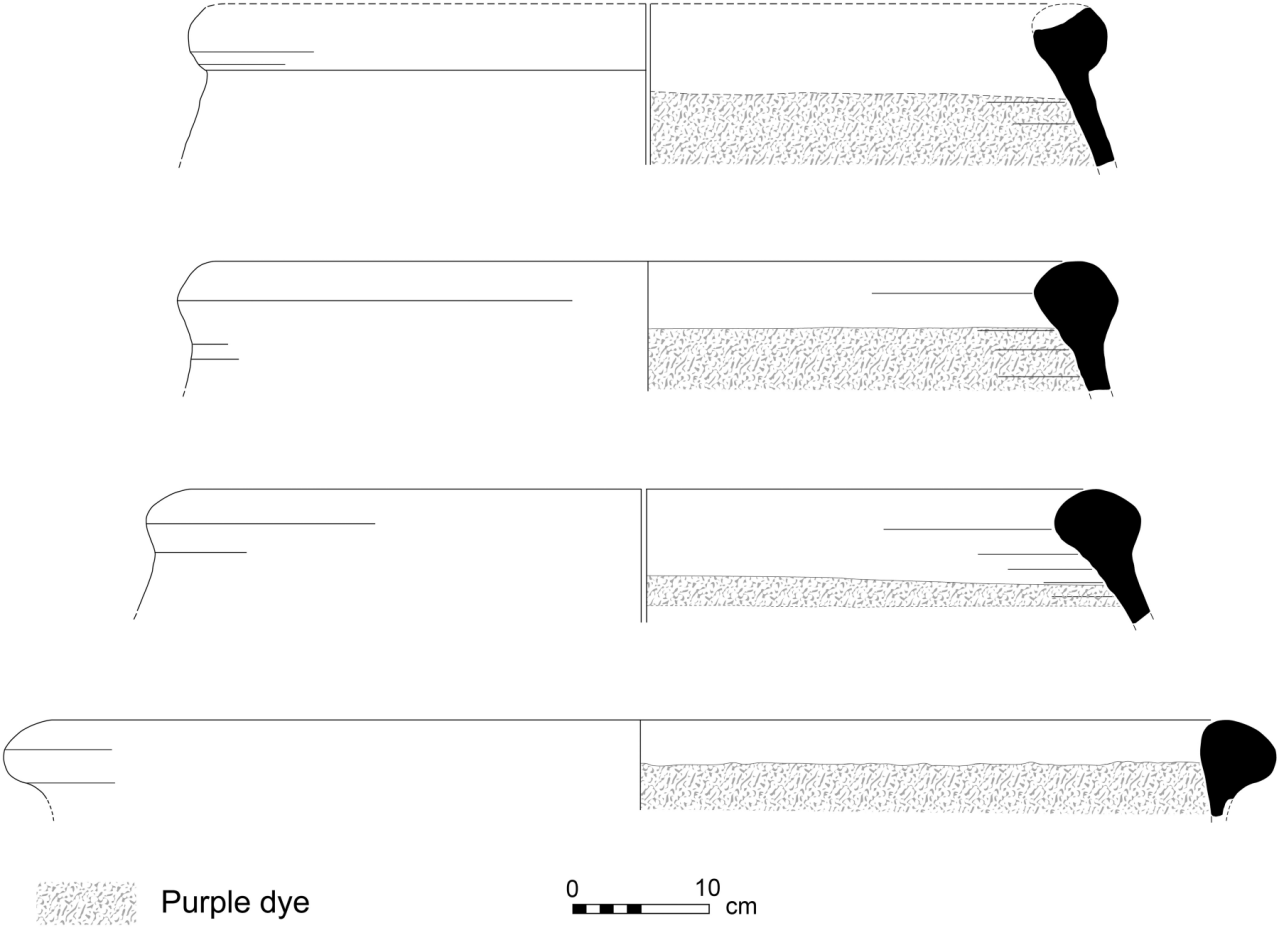
Line-drawings of rims of purple-dye vats with dye remains. Top to bottom: 12B-7005; 6296; 6313; 13A-2020. Drawings by Sapir Haad.

**Fig 5:**
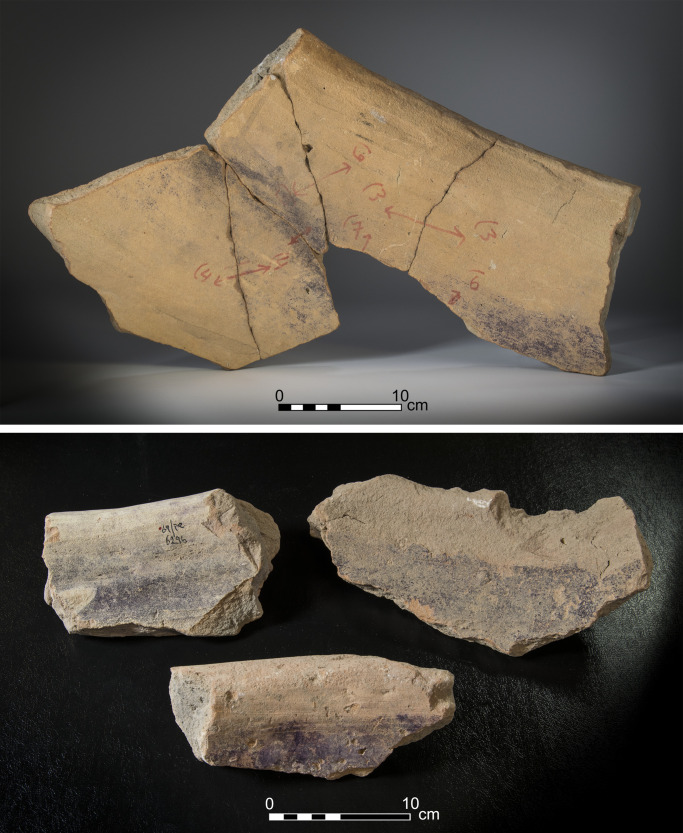
Fragments of vats with short segments of the body with purple dye remains. Upper photo: a restored rim and body fragment of vat no. 7055.1–5. Lower photo: top left: 6296; top right: 12B-7005; bottom: 13A-2020. Photos by Moshe Caine; registration numbers and other markings on the sherds are from the 1960’s–1970’s excavations.

**Fig 6:**
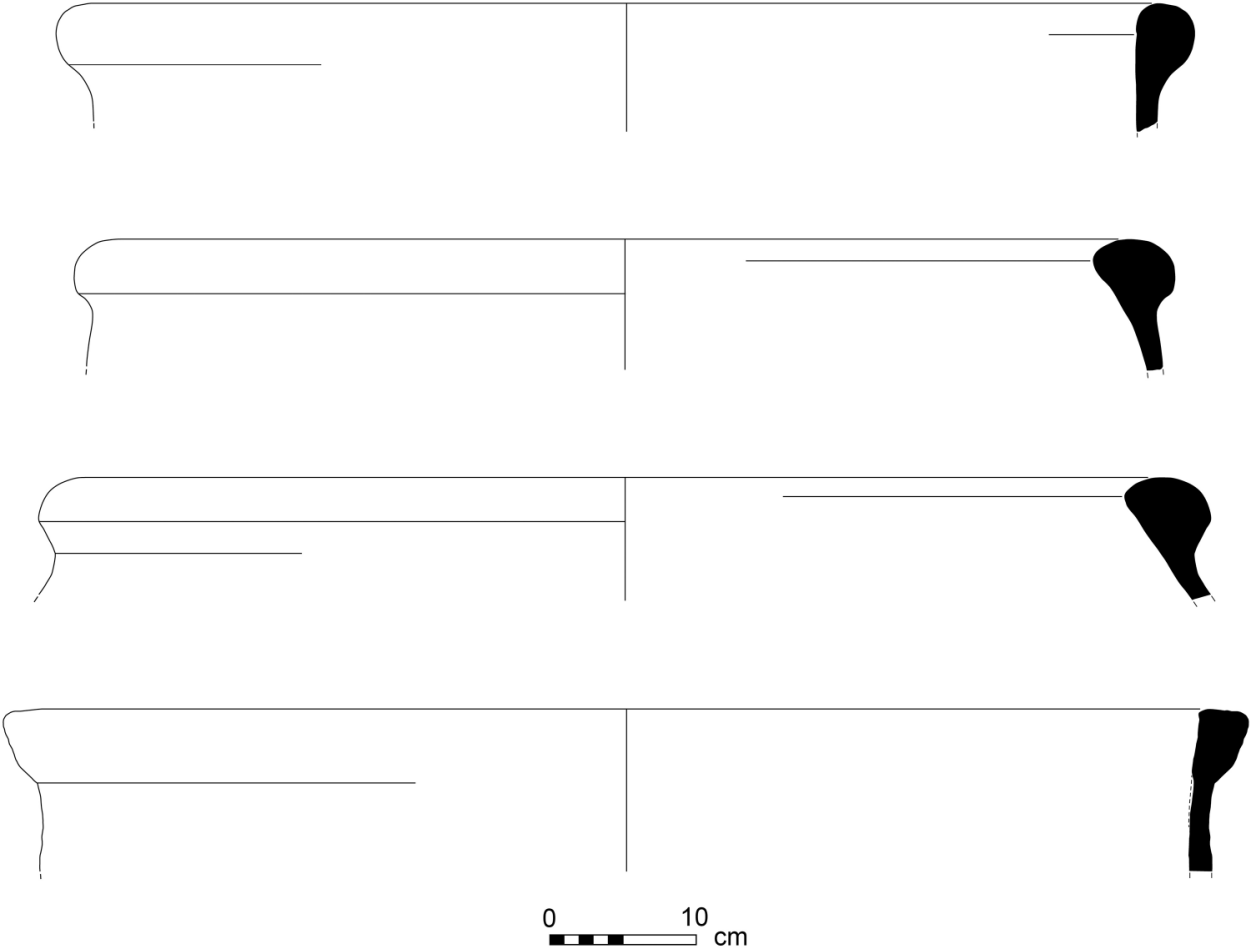
Line-drawings of purple-dye vat rims with no visible dye remains. Top to bottom: 5599; 727; 6270; 5799. Drawings by Sapir Haad.

**Fig 7:**
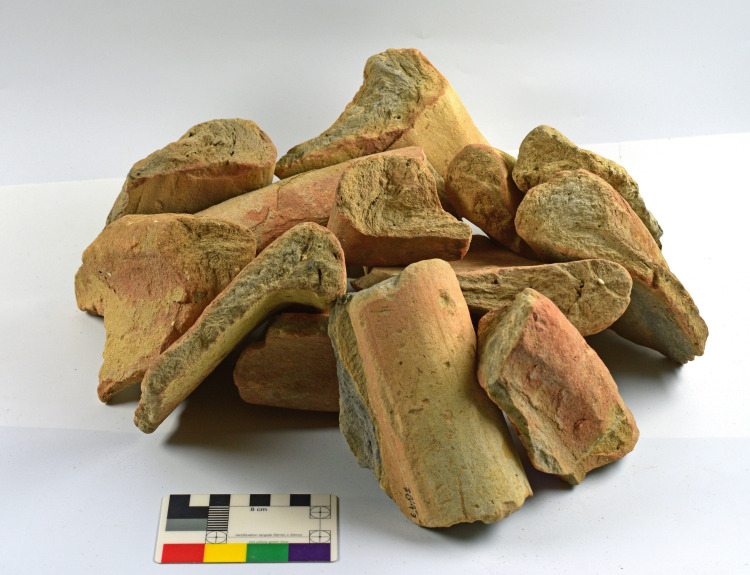
Rims of vats without visible purple remains. Photo by Maria Bukin.

**Fig 8:**
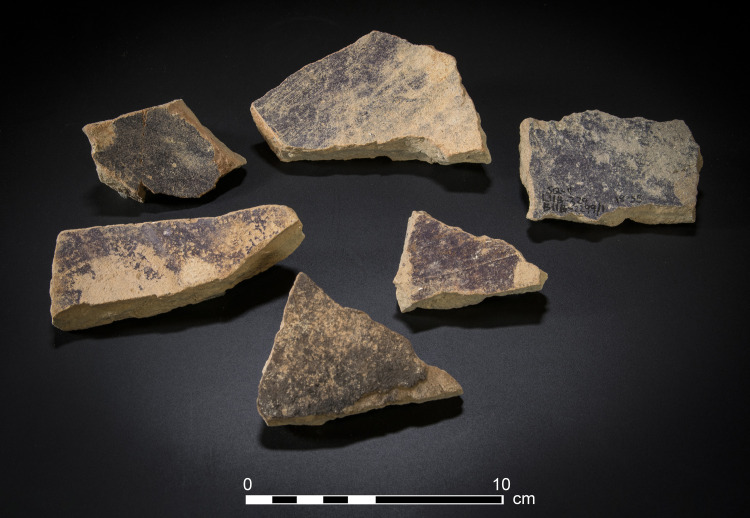
Photo of body sherds of purple dye vats with purple dye remains. From the upper sherd and clockwise: 11B2077/1; 11B2299/1; 5277; 5336; 7238; 2068. Photos by Moshe Caine.

**Fig 9:**
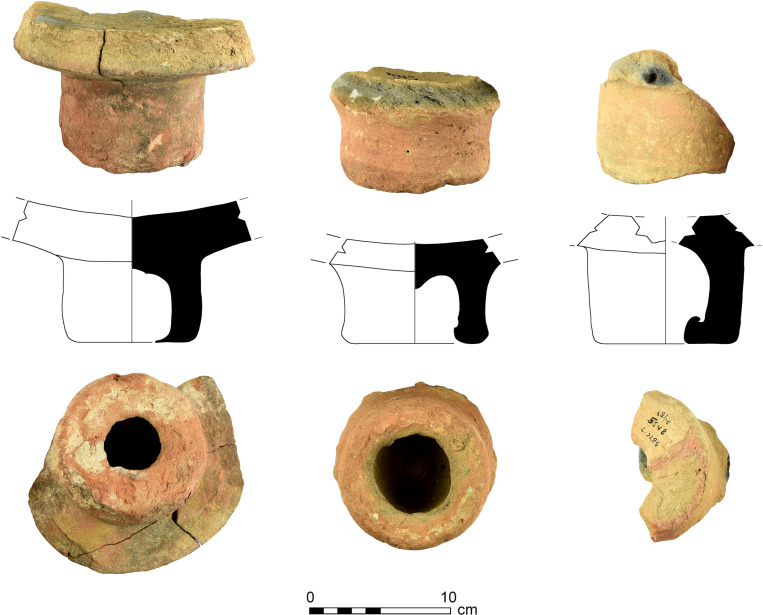
Profile views, section drawings, and bottom views of bases of purple dye vats. Left to right: 6255.5, 8076.10, 5548. Photos by Maria Bukin; drawings by Vladimir Lehem.

In all, at Shiqmona we identified 176 artifacts that can be associated with the production of the dye and/or with the dyeing process (S3 Table). We divide them into the following six main categories:

1) Purple-stained vat rims (N = 7; Figs 4 and 5). The rim fragments belong to massive clay basins produced of coarse-grained material (see below, Ceramic petrography). The vessels’ openings are 60–80 cm in diameter, with thickened rims. As we detail below (Results of chemical analysis), *all* purple stains originate from pigment extracted from Muricidae shells. On one sherd, where a large portion of the body was still attached to the rim, the dye clearly appears as a 10-centimeter wide band on the upper inner portion of the vessel, under the rim (Fig 5). As previously observed [39,55,57], based both on this specific rim and the Tell Keisan pithos [40], this phenomenon is probably due to the fact that only the uppermost portion of the liquid mix in the vats was exposed to oxidization, resulting in a purple ‘band’. Below that band, no oxygen penetrated and the mix remained colorless while the dyeing took place [39,55].

2) Vat rims with no visible staining (N = 31; Figs 6 and 7). These rims are of the same type as Category 1, in shape and in the reconstructed diameter of the vessels, and are made of the same fabric (below, Ceramic petrography). We thus argue that they represent the same specialized vats, possibly not filled to their rims.

3) Body fragments of vats with purple remains (N = 29, Fig 8). These sherds, also produced of coarse-grained fabric (below, Ceramic petrography), are relatively thick (ca. 1.0–1.5 centimeter). Typically, the inner parts of the sherds are completely stained. These fragments probably belong to the uppermost part of the vats (see above and Koren [39] regarding such a vat fragment from Kabri, similarly stained).

4) Vat bases (N = 10, Fig 9). These are heavy, hollow clay cylindrical bases fabricated from the same coarse-grained material as the vat rims and body fragments (see below, Ceramic petrography). The association between the bases and the vat rims was already suggested by Karmon and Spanier [54,55] and we concur, for the following reasons: (1) As mentioned, both belong to massive industrial vessels and share mineralogical attributes. (2) Two bases that also retained part of the vessels’ body (Fig 9: left, and Fig 13) show that these bases formed parts of very large receptacles. (3) The bases are unique, unattested outside of Shiqmona, while the vat rims are also very rare (see more below). (4) There is a contextual association between most of the cylindrical bases and the vat body sherds and rims (Categories 1–3). As expected, the bases are not stained, since the dye mixture at the bottom of the vats was not oxidized (see above, Category 1).

**Fig 10:**
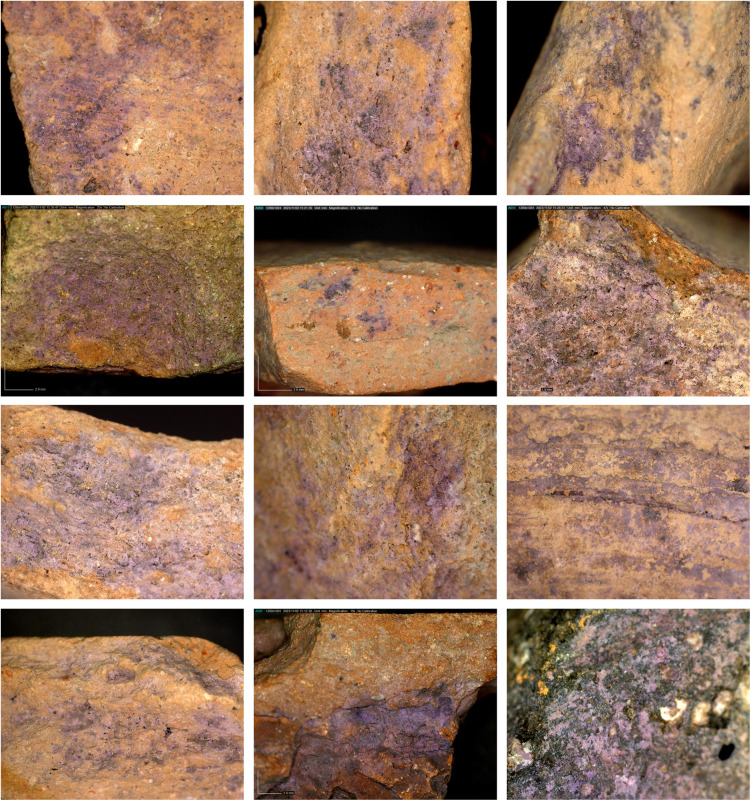
Dino-Lite digital microscope photos of purple-dye residue on miscellaneous pottery vessels. Photos by Golan Shalvi and Maria Bukin.

**Fig 11:**
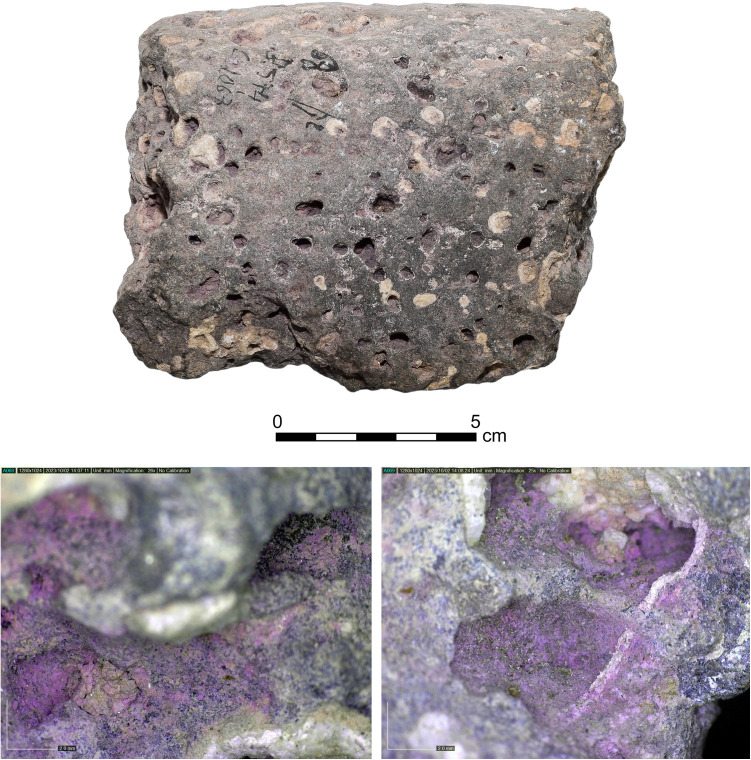
Basalt grinding stone tool 5574 with purple dye residue. Below are the Dino-Lite digital microscope photos of the purple dye residue on it. Photos by Maria Bukin.

**Fig 12:**
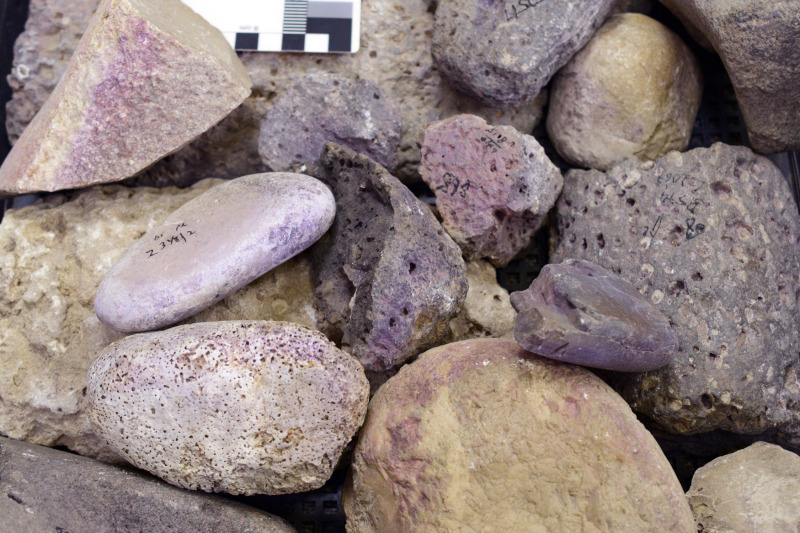
Stone tools with purple dye residue. Photos by Maria Bukin.

5) Purple-stained miscellaneous potsherds (N=82, Fig 10). Beyond the vats, over 80 fragments of purple-stained potsherds exhibit at least some of the following characteristics: (1) The stained areas are small, but clearly observable either visually or under magnification (see below). (2) The distribution of the pigment over the sherds is uneven. (3) The stains appear randomly on various parts of the vessels, including necks, rims, and bases, and occur in diverse locations, including the interior and exterior, and frequently on the fragments’ sections/breaks, indicating that many sherds absorbed the dye after the vessels broke. (4) The fragments are generally smaller and thinner than the body sherds of the vats. (5) Frequently, specific pottery scatters from specific contexts at the site contained multiple stained sherds belonging to various types of ordinary vessels, such as cooking pots, bowls, jars, jugs, and more. Most of these were probably stained secondarily in refuse contexts. This is especially obvious when potsherds revealed stains on their broken sections, indicating that they were stained after breaking (e.g., upper row in Fig 10).

6) Purple-stained stone tools and objects (N = 17, Figs 11 and 12). About 5% of the Iron Age stone artifacts at Shiqmona bear purple stains. The assemblage includes five broken basalt grinding stones and one made of beachrock, two broken basalt bowls (with dye remains also on the surfaces of their breaks), four pebbles that may have been used as hammerstones, one stone weight, one stone rim that seems to belong to a stone vat, and three yet undefined stones. As with the items of Category 5, here too, pigment appears occasionally on the sections of broken stone tools, mainly of basalt. Although this may signify secondary staining of the stone objects, secondary use of the basalt tools, such as for cracking shellfish, cannot be ruled out.

In this paper, we do not address the malacological evidence from Elgavish’s excavation since we could not locate most of the shells. Sieving sediments was generally not practiced in the 1960’s and 1970’s in excavations of historical periods in Israel and indeed the remaining shells show that they were collected manually and selectively—mainly large and ‘nice’ specimens of both Muricidae and other taxa were kept, but also occasionally Muricidae fragments. Minimally, we can say that all three relevant species were present, some with predation holes [41].

### Contextual and stratigraphical analysis: Attributing finds to strata

We attempted to associate the purple dye-related material with the re-evaluated stratigraphic sequence at the best resolution possible. However, some challenges remain. Fifty years ago, the original excavators did not always explicitly address the depositional nature of the various contexts or their relation to architecture, which we reconstructed post-factum. When the objects came from a location with complete ceramic vessels indicating primary deposition, were sealed under architectural elements (such as a wall or floor), or were reconstructed from several joining sherds (assuring minimal re-deposition), the association with specific strata was relatively straightforward. However, several objects originated from ill-defined fills. In these cases, the stratigraphical and architectural attribution was based on correlating the elevations of the finds recorded during the excavation with the architectural elements in their immediate vicinity and on the chronology of the ceramics in the same contexts.

For most of the objects, these criteria were merged successfully, and they could be allocated to specific layers. When an artifact met only two requirements, it was allocated to a stratum with a question mark (S3 Table).

**Fig 13:**
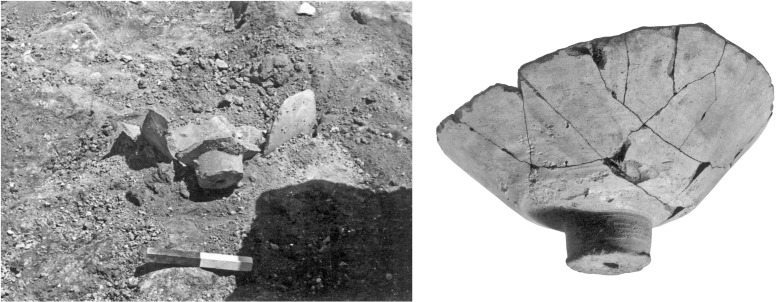
The purple dye vat base from Stratum 11, building B283, basket 6296. Left: photo during excavation (25.8.1969); right; photo after restoration. Unknown photographers (the object itself is apparently lost).

Surprisingly, Shiqmona did not produce any examples of complete or even near-complete vats, not even in the destruction layers of Strata 13, 11, 8, and 7. In Building 283, Stratum 11, for example, a large assemblage of complete ceramic vessels restored from many fragments was excavated near a cluster of loom weights, an olive press installation and tabuns (clay ovens) [53]. Alongside this material, three vat bases were uncovered (including one of the largest at Shiqmona, restored from many pieces; Fig 13) and not far from there, in the same room, two vat rims, one with visible purple coloring and one without. This context had the potential to produce a complete vat. Our only explanation for the lack of such complete examples is that because the shape of the vats was unknown, further body fragments belonging to them were not recognized as such and were discarded with the rest of the so-called non-indicative pottery.

### Calculating the minimum number of individual vats (MNI)

This calculation applies only to Categories 1–4, which are components of purple dye vats. The total number of identified specimens (NISP) is 77 (S3 Table: Categories 1–4). Since the vats were produced from the same fabric (see below), the latter could not guide us in clustering fragments into vessels. When different vat parts were discovered in the same architectural space (room, courtyard, street, etc.), they were considered as one vat. Any number of body fragments from the same space were counted as one vat, but only one base and one rim (or several morphologically identical rims) were attributed to the same vat. For example, if two bases, four morphologically different rims, and 10 body sherds of vats were found in the same room, totaling 16 pieces, the MNI was calculated as 4.

**Fig 14:**
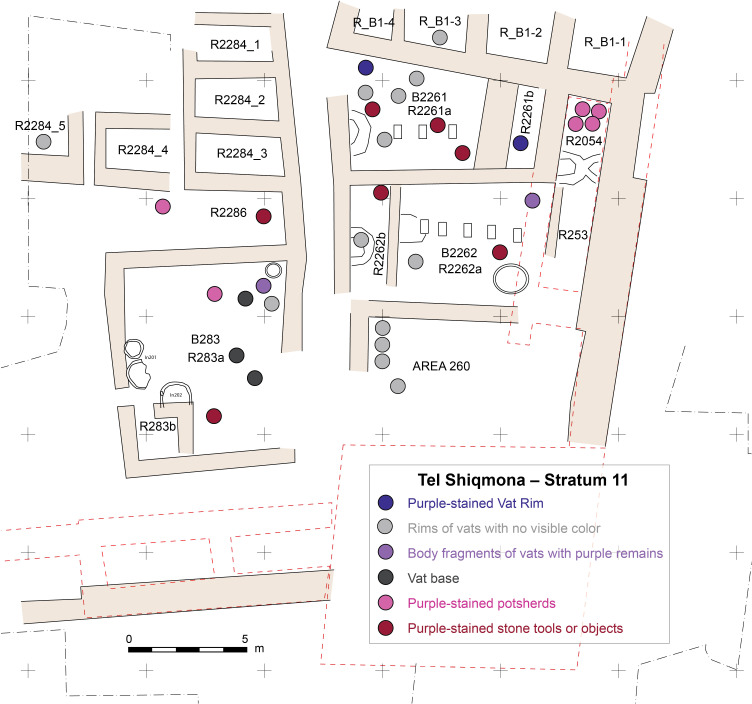
Schematic plan of Stratum 11 with spatial distribution of finds related to purple-dye production. Illustration by Sapir Haad.

To illustrate this, Fig 14 shows the spatial distribution of purple-related artifacts in Stratum 11 (first half of the 8^th^ century BCE). Room R2261a, for example, produced four morphologically different vat rims, three stones with visible purple staining, one stained vat body sherd and possibly another one near it but with a questionable stratigraphical attribution (S2 Table: R2261a; Fig 14). We reconstruct a minimum of four vats in this room, but there could have been more. On the other hand, we cannot know how many served simultaneously during the estimated ca. 50-years-long occupation, if any (for the date and longevity of this stratum, see Shalvi and Gilboa [53]).

Of the vats parts with less certain stratification (marked with question marks in S3 Table) or unstratified contexts, only the rims were counted. Similar rims were counted as one unless depositional considerations indicated that they could not belong together.

### High-performance liquid chromatography (HPLC) chemical analysis

To identify the biological sources of the purple on the artifacts, we used high-performance liquid chromatography (HPLC). This chromatographic method is efficient in separating and identifying compounds in mixtures, and is commonly used in analytical chemistry and biochemistry. The molluskan purple dye comprises a few colorant components, which can be identified by HPLC. Two of them, indigotin (IND) and indirubin (INR), also occur in plants such as woad (*Isatis tinctoria* L.) and the indigo plant *(Indigofera tinctoria* L.), as well as in several species of shellfish. Other components that confer purple color are present only in molluskan dyes: 6-monobromoindigotin (MBI), monobromoindirubin (MBIR), 6,6-dibromoindigotin (DBI), and 6,6- dibromoindirubin (DBIR) [17,58–60]. Other minor dye components, such as isatinoids and indirubinoids, have also been detected in the past in purple pigments [58,61], but since they are present only as traces even in modern purple-dyed artifacts, we do not include them in the analysis.

Previous studies have demonstrated that HPLC can identify the molluskan dyes in the remains of archaeological textiles [17,62,63] as well as on pottery [39,59,62]. This analysis requires only minuscule quantities of the substance and can accurately identify the dye components, allowing for the identification of molluskan dyes with certainty. In some cases, it enables the identification of the specific Muricidae species from which purple was extracted with high probability.

Distinguishing between the three species is, however, not easy because numerous variables such as specific habitat, age, and sex of the snails, the dyeing process, analytical methods employed to record the composition, etc. may affect the measured composition of the purple dye [59,64]. Nevertheless, previous studies [17,39,61,64], showed that it is possible to distinguish *H. trunculus* from the other species based on the relative ratios of the components as seen in the chromatogram. *H. trunculus* contains a relatively high concentration of indigotin (IND) and a high concentration of monobromoindigotin (MBI). In most cases, the amount of DBI is smaller than MBI (DBI ≤  MBI). In contrast, in B. *brandaris* and *S. haemastoma* the DBI compound is the most abundant (over 60%), but the proportion of IND in dyes derived from these two mollusks is low (less than 5%) and cannot always be detected by chromatography. In *Hexaplex trunculus*, the sum of the percentages of IND and MBI exceeds the sum of DBI and DBIR. In other words, IND +  MBI>  DBI +  DBIR. Conversely, in *B. brandaris* and *S. haemastoma* this relationship is reversed: the sum of IND and MBI percentages is lower than the sum of DBI and DBIR percentages (IND +  MBI < DBI +  DBIR). This distinction in chemical profiles provides a reliable means for identifying the biological source of purple pigments in archaeological objects.

Twenty-eight items with apparent purple residue from Shiqmona were sampled and analyzed by HPLC (S3 Table), of which five from Bar’s excavations were previously published [57]. The examined objects include 17 body fragments of vats with visible purple, four purple-stained vat rims, four purple-stained miscellaneous sherds (not of vats), and three purple-stained stone objects (for the protocol of analysis, see S1 File).

### Ceramic petrography of the vats

Ceramic petrography is a well-established analytical method used to determine the mineralogical composition of clay artifacts, identify production techniques and pinpoint the geographical provenance of the materials used in the manufacture of the pottery [65,66]. Petrography is particularly useful for studying pottery produced on oceanic coasts due to its ability to reveal the presence of coastal and marine components in ceramic fabrics. The geology of the coast of Israel varies significantly over short distances due to diminishing quantities of silt originating in the Sahara and Nile-derived quartzitic sand, which decreases northward with distance due to wave erosion. Aeolianite sandstone (locally known in Arabic and Hebrew as *kurkar*) and red loamy soils (*hamra*)—typical of the Israeli coast—vary in their mineralogical composition. Occasionally, marine components such as coralline algae and the shells of Holocene marine microfauna can also be seen in the fabrics, connecting the pottery to its coastal production. In addition, coastal rivers flowing into the Mediterranean contribute different calcareous components, enabling high-resolution petrographic analysis of sediments in the area [67].

The Carmel coast specifically is characterized by calcareous aeolianite sandstone of the upper Pleistocene ridges (*kurkar*) and red loamy soils (*hamra*). These features are surrounded by alluvial sediments that include clay, coastal sand and eroded limestone from the Carmel Mountains [68–70]. Several geological studies revealed that beneath the coastal sand of the Carmel coast lie brown sandy clay units dating to the end of the Pleistocene–beginning of the Holocene, representing a short-lived episode of brackish wetlands [71,72].

We analyzed 34 fragments of vats petrographically (S5 Table). They comprise rims with and without visible purple as well as bases and stained body sherds, and represent a minimal number of 24 vessels.

### Fourier-transform infrared (FTIR) spectroscopy

To supplement the petrographic analysis in determining the mineralogical composition of the ceramic vats and to estimate firing temperatures, FTIR analysis was performed on 11 samples (S5 Table). This builds on well-developed methodologies for the study of archaeological sediments, heated and unheated mudbricks [73,74] and ceramic vessels [75–77]. Ceramic samples were taken from the freshly-exposed section used for the petrographic analysis. The samples were prepared following the conventional potassium bromide (KBr) transmission method [78]. For the detailed protocol of analysis, see S2 File.

## Results

### Artifact database and minimum number of individual vats (MNI)

S3 Table lists all the artifacts that we relate to purple dye production and their stratigraphic attribution. Out of 176 artifacts, 77 are parts of the vats used in the production and dyeing process: rims, body fragments and bases. Among them, a minimum of 29 vats (MNI) could be related to specific strata: two to Stratum 13; seven to Stratum 12; 16 to Stratum 11; and one vat each to Strata 10 to 7. Another nine vessels (MNI) could not be attributed confidently to any specific stratum but unequivocally belong to the Iron Age sequence. The limited extent of the occupations of Strata 10 and 9 (just short of 100 sq m, see [51]), and the fact that Strata 8 and 7 were disrupted by extensive building during the Persian period explain the few vats in these contexts [51,52]. Five of the nine vats with unclear stratigraphic attributions were classified as most likely originating from Strata 7 or 8.

We reiterate that we defined the minimum number of vessels per stratum, and the actual total could be higher, though probably not by an order of magnitude.

### Results of chemical analysis

**Fig 15:**
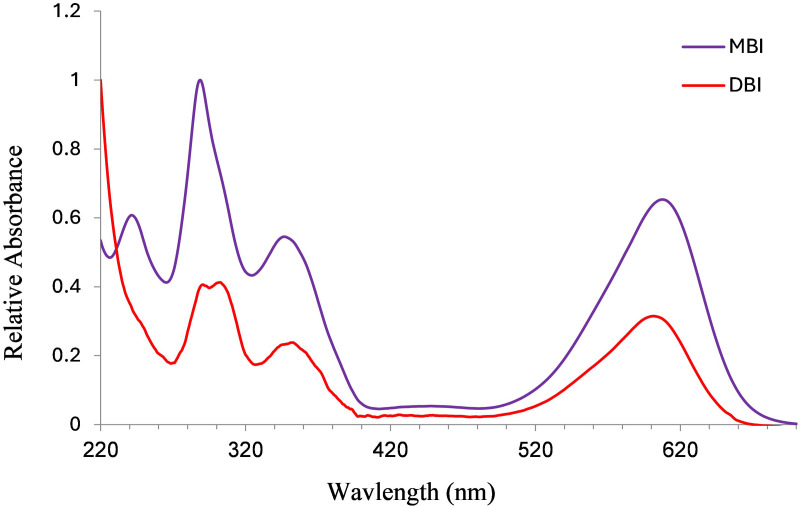
UV–visible spectra of the compounds found in the Muricidae species.

The HPLC analysis revealed that all 28 artifacts examined, namely 25 pottery fragments and the three stone artifacts (S3 Table), were stained with ‘true’ purple dye produced from Muricidae species. All the samples contain at least one of the two molecules that are principal markers of this dye, namely MBI and DBI (Fig 15). Calculating the ratios between the different substances seen in the chromatograms and the identification to species level was conducted for the 20 best-preserved samples (S4 Table).

The relative ratios of the components seen in the chromatograms from these 20 samples indicate that all of them were stained with *H. trunculus* dye: The chromatograms show a relatively high concentration of IND and a high concentration of MBI (IND +  MBI>  DBI +  DBIR (S4 Table). In five samples, a relatively high percentage of DBI and DBIR (above 20%) was also found, which could result from double dyeing using *H. trunculus* in combination with either *B. brandaris* or *S. haemastoma*. Pliny the Elder mentioned this method for obtaining reddish-purple colors (Pliny, Natural History: IX, 62, 137). Another option is that this composition results from *H. trunculus*, including DBI-rich varieties [21,61].

The dominance of *H. trunculus* indicated by the chemical analysis at Shiqmona is compatible with the results of the classification of over the 1,000 Muricidae shells from Bar’s excavations [57] and with the distribution of Muricidae species in archaeological sites across the Mediterranean, as shown in various archaeomalacological studies [16,79–81]. The widespread use of this species is attributed, among other factors, to its broader distribution and greater availability in the Mediterranean compared to the other two. Furthermore, the gland of *H. trunculus* contains significantly more dye than that of the other species (0.9–1.2 mg in *H. trunculus* compared to 0.4–0.6 mg in *B. brandaris* [6,78]), a fact confirmed by our dyeing experiments.

### Results and interpretation of ceramic petrography

**Fig 16:**
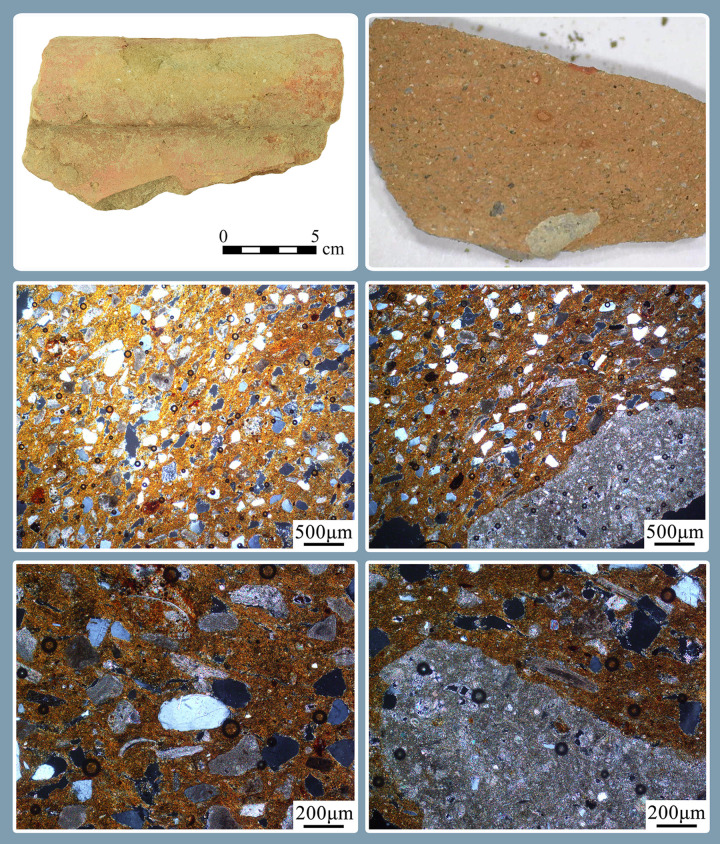
Example of a basin fragment without visible dye. Including a photomicrograph of the fresh break of a fully oxidized section and the petrographic thin section at X50 and X100 magnification under XPL, showing a calcareous matrix, quartz sand, shell fragments, and foraminiferous chalk (S5 Table: no. 28).

Perhaps not surprisingly, all 34 samples analyzed belong to the same petrographic group, representing the very well-known production of the Carmel coast [82]. The clay is carbonatic, optically active and silty (~10%), tan in PPL, with some iron oxides. The silt is mostly quartz but also contains some feldspars and shell fragments. The inclusions consist mainly of well-sorted sub-angular quartz sand (~30% up to 200µm), and poorly-sorted limestone, ranging in size from fine to coarse. Also seen are foraminiferal chalk, *kurkar* fragments and tuffs. Some of the samples also contain fragments of beachrock and Holocene microfauna typical of shallow marine waters, such as coralline algae, mollusks, foraminifera and ostracods (Fig 16; S5 Table).

This petrofabric group is well known from pottery and mudbricks as early as the Neolithic/Early Chalcolithic around ‘Atlit Yam, ~ 12 km north of Shiqmona [83], and it is attested at least until the Byzantine period and possibly later [84]. During the Iron Age, this group is particularly well-known from the extensive ceramic production at Dor and Shiqmona for a large variety of calcareous pottery vessels [85,86]. Based on their mineralogical characteristics, the vats could have been produced anywhere on the Carmel coast from Dor to Shiqmona, possibly as far north as Tell Abu Hawam.

**Fig 17:**
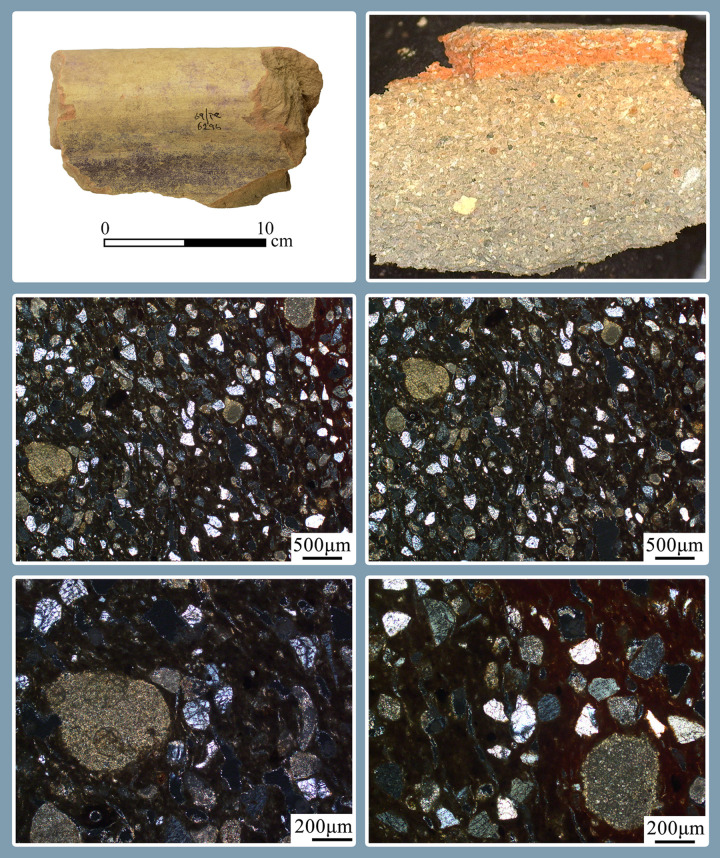
Example of a basin with a visible layer of dye. Including a photomicrograph of the fresh break of a section with a black core and the petrographic thin section at X50 and X100 magnification under XPL, highlighting a dark matrix, quartz sand, and corroded calcareous fragments (S5 Table: no. 21).

In cases where the thin sections were cut through the interface between the surface of the vats and the layers of dye, the color of the ceramic matrix was dark (Fig 17), indicating the partial dissolution of the calcareous components in the basins, a phenomenon that requires further investigation.

### Results of FTIR

**Fig 18:**
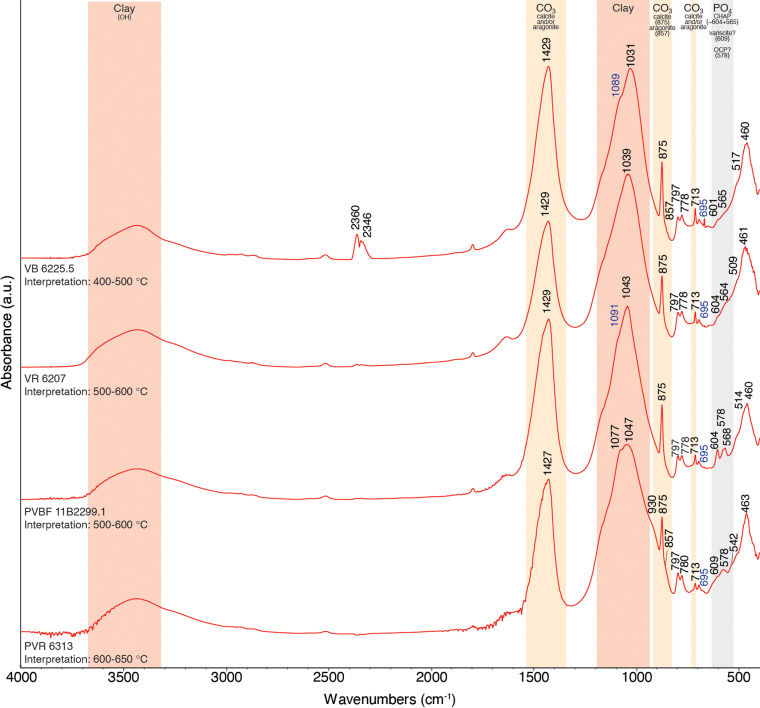
Representative FTIR spectra and interpretation of firing temperature of select sherds from Shiqmona.

Representative FTIR spectra of the Shiqmona vats are presented in Fig 18. More details, including all spectra, are available in the S2 File and S5 Table. Overall, the spectra were overwhelmingly similar and characterized by a composition of low-fired clay, calcite, quartz, and phosphate minerals (of the latter, for the most part, carbonated hydroxyapatite). Some organic material is present in all samples, but at present we cannot determine its source. The absence of absorbance bands indicative of structural water (hydroxyls bound in clay minerals) in all samples in the 3400–3600 cm^-1^ region, the position of the main clay bands between 1039–1043 cm^-1^, and the absence of the band at 913 cm^-1^, determines that most of these basins were fired or heated to temperatures of approximately 500–600 °C (cf. [73,74]; for the significance of this observation see below). The presence of calcite in all samples, which begins to decarbonate around 600–650 °C [75] provides an upper limit for the firing temperature of these vessels.

Two samples (6255/5 and 5548) were fired at lower temperatures between 400–500 °C, based on the location of the main clay peak at 1031 and 1035 cm^-1^ respectively, and both contain traces of aragonite, likely related to mollusk inclusions in the ceramics (see the petrography section). One sample (6313) was likely heated to slightly higher temperatures of 600–650 °C, indicated by the shifting of the main clay peak to 1047 cm^-1^.

## Summary and discussion

Purple production at Shiqmona was practiced continuously from Stratum 15 to Stratum 7, ca. 1100–600 BCE. From Stratum 13, the 9^th^ century BCE, a specialized industry is in evidence, on a hitherto unattested scale. Shiqmona is also the only site where actual purple-dye workshops have been excavated, which operated for hundreds of years.

In all, we recorded 176 artifacts related to purple dye production, among them 135 items stained with purple. Twenty-eight samples, of all the categories of stained artifacts we defined (i.e., other than unstained bases and rims), underwent chemical analysis, which proved that they were dyed with Muricidae species. This, we argue, permits us to cautiously, but confidently extend this conclusion to all other 107 stained objects. This is by far the largest such assemblage ever uncovered around the Mediterranean in any period, by two orders of magnitude.

Out of the 176 artifacts, 144 are attributable to specific strata: 14 to Stratum 13; 12 to Stratum 12; 33 to Stratum 11; six to Stratum 10; 34 to Stratum 9; 36 to Stratum 8; nine to Stratum 7 (see S3 Table). Production of purple is attested already in Strata 15/14 but vats are clearly present only from Stratum 13 onward. Counting MNI, the artifacts represent a minimum of 38 production vats from the Iron Age. Considering that in all the excavations combined, about 30% of the extent of the tell has been excavated, the number may have been much higher, and we repeat that this is a minimum number.

The petrographic and mineralogical analysis indicates that the vats were produced on the Carmel coast, and most likely indicate local production at Shiqmona, where beachrock outcrops are exposed at the foot of the tell. The low firing temperature, ranging between 500–600 °C, of all ceramic vats, revealed by FTIR is surprising, as firing below 600 °C barely allows the clay to sinter (i.e., when the particles bond together giving the material strength and durability, [87]). Low-fired ceramics also remain somewhat porous and are not completely waterproof. In comparison, amphorae and other ceramic vessels produced during this period from the same Carmel coast raw materials as the basins, were fired to 750 °C to 900 °C [85]. We assume that the choice of relatively low temperatures is due to constraints related to the easily available raw material and production of the vats themselves, and not to the dye-production procedures, see the commentary in Supplementary S2 File. Importantly, beyond the specific shape of the vats, the petrographic and FTIR analyses point to a specialized and remarkably consistent vat production tradition that lasted for half a millennium.

As shown in the example in Fig 14, the vats are present in all the excavated architectural complexes, except, for example, small storerooms and alleys. This indicates that all the excavated buildings served the purple industry, probably both for manufacturing the dye and for dyeing process (see below).

About 50% of the stained objects, including potsherds and several stone items (of Categories 5 and 6), cannot be associated directly with the dye production or dyeing. Many of them were probably tinted secondarily in refuse contexts, often after they broke. This likely attests mainly to the intensity of production and its longevity throughout the Iron Age, discussed further below. However, others may have served as tools in the purple chain of production, a possibility that requires further investigation.

To provide some sense of the scope of production: In Stratum 11, of 33 purple-related artifacts (S3 Table), we calculated a MNI of 16 vats. Even if we ignore the very likely possibility that the unexcavated 70% of the tell conceal many more vats, this means that about 20 vats were active at some point during a phase lasting approximately 50 years [53]. Surely, such large containers were not replaced every year or season, but we do not know how many of them may have functioned concurrently.

### The purple dye production vat: Connecting the pieces

The conspicuous rims with purple residues, some also preserving parts of the vessel’s body and the unique bases that we associate with them, allow us to roughly define the shape of the vessels used as containers for the dye mixture. (For the association of rims and bases, all belonging to unparalleled uniform massive industrial vessels, see Category 4 and the results of the petrographic and mineralogical analysis.) The thickness of the purple-stained body sherds of Category 3, as well as their mineralogical composition, suggests that they, too, were part of these massive vessels.

**Fig 19:**
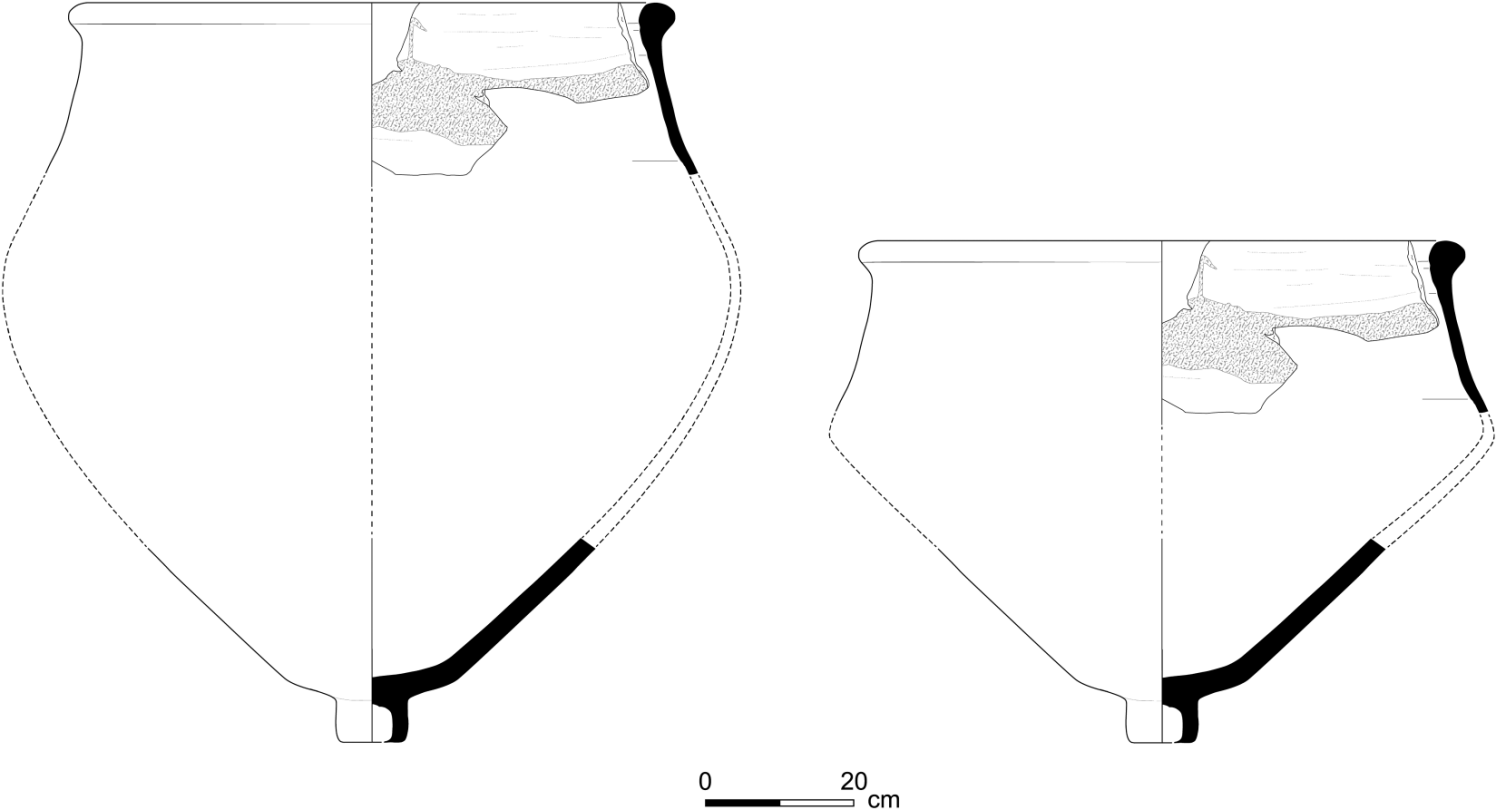
Proposed reconstructions of the purple-dye vats. The reconstruction is based on rim no. 7055 and base no. 6255. Illustrations by Sapir Haad.

Fig 19 shows a suggested reconstruction of the general shape of the vats, combining the largest preserved rim with purple dye remains (Fig 5, upper photo) and the base with the largest vessel profile attached to it (Fig 13). We offer two possible reconstructions, but we opt for a shape similar to the larger one (left), since any smaller option (right) would create an uncommon carination in the vessels’ walls. Notably, in the only other case in the Levant where a complete container used for purple dye production was unearthed—at early Iron Age Tell Keisan—the vessel utilized was a ca. one-meter tall pithos with a wide orifice (see Pl. 69:1 in [40]).

We thus reconstruct vats with an opening of about 70 centimeter, and with walls 1 cm thick or slightly more (thicker near the folded rim and the base). The maximum diameter and height in our preferred reconstruction are about 90 centimeter. The volume of the clay vat itself in Fig 19 (tara) would be 40 liters, calculated using the Pot Utility software [88]. The specific gravity of the vats’ clay is ca. 1.7 kg. The large version in Fig 19 would thus weigh about 68 kg when empty.

The vats could hold about 350 liters, calculated from the bottom to the level where purple is preserved below the rim. This means about 350 kg for the purple solution in the vat, assuming that it mostly comprised water. A vat would weigh about 418 kg when full. The vessels may have been somewhat taller or shorter, wider or narrower, but nonetheless, Shiqmona provides the first clear indication of the type and shape of a rather standard clay vat used for nearly five centuries.

**Fig 20:**
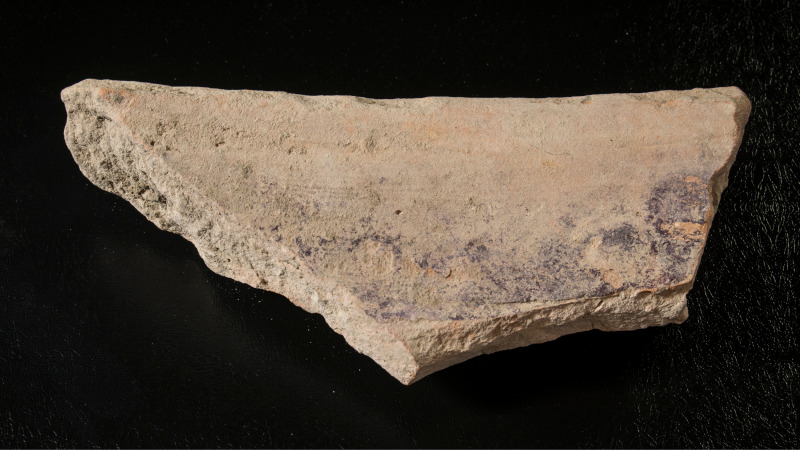
Purple-stained vat rim from Dor. Photo by Moshe Caine.

Parallels to these specialized vessels are rare, but not unknown. Similar rims of comparable size, occasionally with purple remains, have been found at a few coastal or near-coastal Iron Age sites in the Southern and Central Levant. Examples occur at nearby Dor in a mid-7^th^ century BCE refuse context (stained; Fig 20); and the fortified enclosure of Tel Kabri on the plain of Galilee in Stratum E2, at the end of the 7^th^ century BCE (stained; dye analyzed by Koren [89]). Nine identical rims, but without reported staining, were found at Sarepta in south Lebanon during J. B. Pritchard’s excavations in the early 1970’s. They belong to Strata D1–B, generally dating to the 8^th^–7^th^ centuries BCE, but mainly to Stratum C1 of the end of the 8^th^ century (Pl. 38:6, Type RR-3 in [90]; for the dates, see [52,91]). Recently, a few identical rims were found at Tell el-Burak in Lebanon, just north of Sarepta, also in late Iron Age contexts (for example, [92,93]; for the site and economic activities there in the late Iron Age, see [94]). All these vessels indicate that a rather standard large vat was used in the purple dyeing process during the Iron Age, at least from south Lebanon to the Carmel coast, but perhaps only as part of large-scale operations. These vats had no ceramic lids; nothing suggesting such lids has been unearthed in any Iron Age site along the coasts of the East Mediterranean. However, the need to control the oxygen and the lightning conditions in the vats probably necessitated using perishable organic covers (e.g., made of wood, leather, wool, etc.), which did not survive.

**Fig 21:**
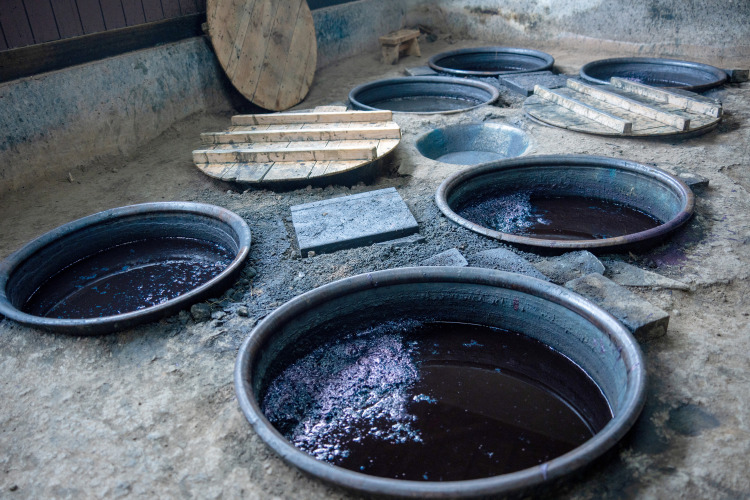
Indigo dye workshop in Chikugo, Japan. Photo by Marisa Tashima (United Native Acumen [UNA] Laboratories).

Although simpler, traditional indigo dyeing might provide an ethnographic analogy to the Shiqmona vat dyeing since it involves similar chemical processes, including reduction and oxidation, and is still practiced today in different parts of the world [6,95]. Indigo dyeing is generally conducted in closed containers so the oxygen supply can be controlled. In Uravakonda (India), for example, dyeing is performed in ceramic vessels sunk into the floors and covered with basket lids. This setup allows for the control of oxygen exposure and prevents insects from entering the dye solution (information provided by Gopal Kanchibhotla [96]). Similar practices were recorded in Oman, where large ceramic vats were sunk into floors indoors and covered with wooden lids [97]. In Chikugo Province, Japan, large containers akin to the shapes we reconstruct in Shiqmona are used for dyeing, almost completely sunk into the ground and covered with circular wooden lids (Fig 21) [98].

Such large containers with relatively minuscule bases could not have been freestanding. We assume that they were not supported by tripods or the like (clay or metal), since there is no evidence for such objects among the vast assemblage of pottery or among hundreds of metal objects/fragments at Shiqmona. Such devices are also unattested more broadly in the Southern Levant during the Iron Age. We conclude that the vats must have been sunk into the ground. However, if they were sunk entirely, complete or partially-complete vessels should have been preserved. This is the case, for example, with jars sunk in the olive-oil press installations at Shiqmona, for collecting the oil [51,53]. Stratigraphic analysis also reveals no indication of pits large enough for these vessels. Finally, while not necessary to produce purple dye [16], it would occasionally have been beneficial to heat the vats during the production process, as described by Pliny and reported by modern experimenters [21]. Burying the vats entirely would cool their contents below room temperature and largely prevent the manipulation of the thermal conditions of the mix in the vats. Therefore, we reconstruct that the vats were only partially sunk into the ground, and the cylindrical bases served to stabilize the slightly buried vats by anchoring them to the ground.

Having said that, we have no evidence that the production of the dye at Shiqmona involved heating. Notably, no soot or other marks of fire were found on the vats and no appropriate heating installations were uncovered. The need to heat the dye concoction would inter alia depend on the season of production, especially in the very warm Southern Levant. An analogy may be found in the traditional indigo dyeing mentioned above. The indigo solution is usually heated at a moderate temperature (around 30–50˚C) for a few days until it turns yellow, indicating the leuco (soluble) form and the completion of the reduction process [6,21,57]. In some regions, however, no heating is conducted, and dyeing is instead carried out on hot days (for example, in Iran [6]).

Be that as is may, heating the solution would not necessarily be performed by applying direct heat but could be achieved by adding warm/hot water.

Finally, we argue that at Shiqmona, the vats served for both the production of the dye and the subsequent dyeing of fibers and/or fleeces. The size and opening of the vats would have allowed the dipping of the fleeces or fibers into the vats. Given their substantial weight when full, it is unlikely that the vats were intended to be moved, nor could they be tilted. Producing the dye in these very large vessels and then transferring it to other containers for dyeing (at Shiqmona or elsewhere) does not seem to be a plausible reconstruction of the process. Decanting the dye with some smaller vessels would undoubtedly result in at least some loss of the precious substance. For the same reason it would be illogical to transfer the dye to another container for dyeing. Therefore, we conclude that the entire manufacture, from harvesting the snails to dyeing, was conducted at the site, and that dye-production and dyeing were conducted in one container – apparently a rather efficient process.

By the distribution of the clay vats comparable to those at Shiqmona (above), the ‘double-function purple vat’ technique seems to have been practiced at other Iron Age sites in the coastal Levant, but we cannot say whether it was the sole technique for purple dye-production. No similar vats are attested, for example, in the subsequent Babylonian and Persian (Achaemenid) periods, between the 6^th^ to 4^th^ centuries BCE. At Dor, a 3^rd^-century BCE dye-production installation comprising two pits with an interconnecting channel [99] exhibits an altogether different method.

The spatial distribution of the vats is also of significance. As mentioned above in passing and illustrated in Fig 14, the vats were clearly found within buildings, not in the alleys (and not in the small store-rooms). This would allow for the regulation of light, air (and wind) during the production process. This contradicts some prevailing assumptions that the accompanying odor would force purple-dye production outside settlements, or at least downwind (see discussions in [16,32,54,100,101]). This is obviously not the case at Shiqmona. The reason is clear, as we already argued above: Shiqmona was a specialized production facility, not a settlement per se. The stench must have been especially acute when the site was enclosed by a defensive wall in Strata 13–12.

### Future prospects, Shiqmona and beyond

Looking at Shiqmona in particular, further chemical and mineralogical analyses should be applied to the vat fragments to investigate other organic and inorganic components of the dye mixture and to reconstruct production techniques such as heating or controlled reduction and oxidation. In the field, we also started to conduct further, higher-resolution excavations to provide data unavailable from the old excavations, including systematically dry and wet sifting sediments and flotation of specific contexts to provide, inter alia, meaningful and quantifiable assemblages of plant remain, bones and shells/shell fragments. High-resolution faunal data, especially micro-fauna, alongside new malacological and botanical data, may shed light on the possibility of seasonal occupations or production at the site.

As for the purple dye production in the Eastern Mediterranean and perhaps in other Mediterranean regions as well, Shiqmona offers a few new insights:

1. Regarding the organization of production, at least from the 9th century BCE—Stratum 13 at Shiqmona—large-scale purple dye industry was in existence. We use the term ‘industry’ considering the scale, the concentration of production facilities, and the specialization evident at the site (cf. [102]), thus also the use of ‘factory’ in our title. Most likely, Shiqmona was not the only purple-dye production center and production was also be located within towns and cities, as was the case at Dor (see also [32]). Whether other specialized centers for purple-dye production and dyeing existed in the Iron Age Levant remains to be seen.
2. ‘Location, location, location’. As we mentioned in the introduction, Shiqmona was an excellent location for the industry since its abrasion platforms provided one of the best habitats for Muricidae along the South Levantine coast. While systematic research has yet to be conducted to explain this phenomenon, for the time being we base the latter statement on the personal experience of the authors and other researchers specializing in the maritime aspects of archaeology and marine biology along the Carmel coast: snorkeling or diving near Shiqmona is the easiest and fastest way to observe many Muricidae (example in Fig 3). Once production was established, it was sustained for centuries, surviving through several geo-political upheavals and many calamities, including total destruction events. Production ended only when the Babylonian armies annihilated the entire regional economic apparatus and nearly every major site in the Southern Levant, after which a century-long hiatus in occupation at Shiqmona is evident [103]. All of this suggests that locating large-scale and long-lived purple-producing sites in the future should start by identifying favorable marine ecosystems.
3. As already mentioned, we argue that at Shiqmona the entire ‘purple process’, from the collection of the shellfish to dyeing—most probably of wool [17,104] was conducted at the same place (for diverse possibilities in several periods, see [23,24]). The large designated clay vats are the only containers at the site associated with this process, and additionally, no significant installations such as water channels, cisterns, rock-cut pits, heating installations etc. on the tell or around it can be associated with purple-dye production or with dyeing. These, indeed, were seemingly not required, since both dye production and dyeing were conducted in the vats. Regarding other stages of producing purple-dye textiles we currently have too little information: though nearly every Iron Age stratum at Shiqmona produced spindle whorls and loom weights [105], we cannot confidently associate them with the production of purple-dyed items.
4. Large-scale purple dye manufacture was a messy business. Shiqmona clearly reveals that large-scale dye manufacturing and dyeing generated significant waste. Dye would adhere to clay and stone artifacts in the production areas and in designated refuse areas. Since, as demonstrated, the stains are easily observable on many (parts) of the objects, these suggest the types of artifacts and staining patterns we should anticipate to find, or look for in other production sites. Indeed, the original number of stained objects must have been much higher, since most of the stained clay and stone items (more than 100) were identified among rims, bases and decorated sherds retained during ‘pottery readings’, after which the rest of the pottery was discarded in both Elgavish’s and Bar’s excavations (as is still common on many excavations in Israel). Based on our experience in Israel, the total reserved pottery usually comprises about 7% of the total excavated potsherds. Finally, we identified most of the stained items in the laboratory with regular daylight, and specialized lighting conditions (such as multispectral imagery) would probably have revealed more examples.
5. The vat fragments at Dor, Shiqmona, Tel Kabri, Sarepta and Tell el-Burak suggest that between the 9^th^ and late 7^th^ centuries BCE, at least from the Carmel coast to south Lebanon, containers of similar shape and size were used for large-scale production of purple dye. Recognizing fragments of such vessels, especially their distinct rims and bases, may enable the identification of more production sites. This may be the case also for nearby regions that during the Iron Age were connected with the coastal Levant through multiple channels of communication, such as Cyprus, the Syrian coast and sites of the Phoenician diaspora [106–108].

We do not put Shiqmona forward as a ‘type site’. Undoubtedly, production technologies, spatial and social organization of production and so forth varied across time and in accord with specific environmental and cultural contexts. Production must also have occurred on smaller scales, for short periods of time, and likely also ad hoc. Shiqmona, however, provides the first comprehensive benchmark for assessing various aspects of specialized purple dye production and dying in a concrete and well-defined timespan of the Levantine Iron Age.

### Authorization and further information on samples

All necessary permits were obtained for the described study, which complied with all relevant regulations (the Israel Antiquities Authority and the National Maritime Museum in Haifa).

Most of the purple stained pottery is temporarily housed at The Zinman Institute of Archaeology at the University of Haifa, but they will be returned to the Israel Antiquities after our study. The registration numbers of all artifacts analyzed in this paper appear in the Supplement.

## Supporting information

S1 File Protocol of HPLC analysis. (DOCX)

S2 File Protocol for FTIR analysis, detailed spectra of the samples and commentary. (DOCX)

S1 Table Sites with direct evidence regarded as indicating dye manufacturing during the Bronze and Iron Ages around the Mediterranean, mainly following Reese forthcoming 2025. Abbreviations: MB=Middle Bronze Age, LB=Late Bronze Age, IR=Iron Age.
(DOCX)

S2 Table The Iron Age stratigraphic/chronological sequence at Tel Shiqmona. (DOCX)

S3 Table List of artifacts related to the purple dye industry from Tel Shiqmona. Abbreviations: PVR = purple-stained vat rims, VR = vat rims with no visible staining, PVBF = body fragments of vats with purple remains, VB = vat bases, PSH = purple-stained miscellaneous potsherds, PST = purple-stained stone tools and objects.
(XLSX)

S4 Table Results of the HPLC analysis; the ratios of the components that were identified at 554. Abbreviations: PVR = purple-stained vat rim, PVBF = body fragment of vat with purple remains, PSH = purple-stained miscellaneous potsherd, PST = purple-stained stone tools and object. Items marked with an asterisk are from Bar’s excavation and were published in Sukenik *et al.* 2017.
(DOCX)

S5 Table List of artifacts analyzed by ceramic petrography and FTIR. Abbreviations: Cl (a) =  altered clay, Ca =  calcite, Qtz =  quartz, P =  carbonated hydroxyapatite, A =  aragonite, Org =  organic material; PVR = purple-stained vat rim, VR = vat rim with no visible staining, PVBF = body fragment of vat with purple remains, VB = vat base.
(XLSX)
