# Supplementary material for: Tel Shiqmona during the Iron Age: A first glimpse into an ancient Mediterranean purple dye ‘factory’
Source: PLoS One. 2025 Apr 16;20(4):e0321082. doi: 10.1371/journal.pone.0321082 (PMC12002455; doi:10.1371/journal.pone.0321082)
Supplement: S1 File — (DOCX) [file pone.0321082.s001.docx]

**S1 File: High-performance liquid chromatography (HPLC) analysis protocol**

**Detailed method:**

The dye substances were analyzed using high-performance liquid chromatography (HPLC) with a diode array detector (HPLC-DAD) at the HPLC Unit of the Mina and Everard Goodman Faculty of Life Sciences, Bar-Ilan University. The analysis employed EZ Chrom Elite v3.2.1 software and a Hitachi LaChrom Elite Chromatography system. The experimental setup consisted of an L-2130 binary pump, an L-2200 autosampler, an L-2300 column oven maintaining a constant temperature of 30 °C throughout all analyses, and an L-2455 diode array detector configured to acquire chromatogram spectra across the 200–700 nm range. Chromatographic separation was performed on a GraceSmart RP18 column (5 μm particle size, 250 mm × 4.6 mm internal diameter). Each analysis yielded a characteristic chromatogram, with color components identified based on their specific retention times (Rt) and distinctive absorbance spectra, including the wavelengths of maximum absorbance (λmax) in the UV-visible spectrum.

**Extraction method**

Samples weighing 3 mg were scraped from the purple surface with a scalpel and placed in an Eppendorf test tube, with 150 μL of dimethyl sulfoxide (DMSO). DMSO is a powerful solvent that can efficiently dissolve blue dye substances (cf., Michel et al. 1992, 147; Koren 2008, 384; Sukenik et al. 2021). Each sample was heated to 95 °C in a bath for 10 min. Once the solution turned blue, it was separated from the settled sediment and transferred to a clean Eppendorf test tube. Finally, the sample was centrifuged for 5 min at 12000 g, and 25 μL of the supernatant was injected into the HPLC column.

The mobile phase for the protocol used in this study was made up of A: phosphoric acid 0.5% (w/v); B: methanol, and C: H_2_O. The flow rate was held at 1 ml/min, and the injection size was 25 μL. Gradient elution conditions are tabulated in S4 Table.
