# Supplementary material for: Tel Shiqmona during the Iron Age: A first glimpse into an ancient Mediterranean purple dye ‘factory’
Source: PLoS One. 2025 Apr 16;20(4):e0321082. doi: 10.1371/journal.pone.0321082 (PMC12002455; doi:10.1371/journal.pone.0321082)
Supplement: S2 File — (DOCX) [file pone.0321082.s002.docx]

**S2 File:** **Protocol for FTIR analysis, detailed spectra of the samples and commentary**

(Prepared by Zachary C. Dunseth and Paula Waiman-Barak)

**Detailed materials and methods for FTIR analysis**

Approximately 10–30 μg of ceramic material sampled from a fresh break was first ground using an agate mortar and pestle and mixed with approximately 30 mg of KBr. Each pellet was analyzed between 4000 and 400 cm^-1^ at a 4 cm^-1^ resolution using a Thermo Scientific Nicolet iS5 spectrometer with Omnic 9.3 and Macros Basic software (Thermo Scientific). The first difference second derivative function in Omnic 9.3 was used to distinguish and confirm the location of indicative bands in shoulders and broad absorbance bands (following [1]). Spectra were compared to the extensive Kimmel Center for Archaeological Science, Weizmann Institute reference library (<https://centers.weizmann.ac.il/kimmel-arch/infrared-spectra-library>), and published experimental (e.g., [2–3]) and archaeological data (e.g., [4–6]).

**Spectra and detailed interpretations begin on the following page.**

**Stratum 15a**

**Purple Vat 11B-2299.1 (PVBF)**

This spectrum is characterized by mid-to-low-temperature heated clays, calcite, quartz, phosphate (carbonated hydroxyapatite) and organic materials. The main clay absorbance band is at 1043 cm^-1^, while absorbance bands of structurally bound hydroxyl in clay minerals (in the region from 3600-3400 cm^-1^) are absent, and the band at ~913 cm^-1^ is absent. Following experimental data, this suggests the clay minerals in the ceramic were exposed to approximately 500-600 °C (cf. [2]). The absorbance bands at 1429, 875 and 713 cm^-1^ are indicative of calcite. Quartz is indicated by shoulders at ~1080 and ~1170 cm^-1^, the doublet at 797 and 778 cm^-1^, the band at 695 cm^-1^ and the shoulder at 514 cm^-1^. The presence of carbonated hydroxyapatite is indicated by absorbance bands at 604 and 563 cm^-1^. A shoulder at ~578 cm^-1^ is not confidently identified. Organic material, possibly contaminants, is visible in the region from ~3000-2800 cm^-1^.

Mineralogy: Clay (heated, 500-600 °C), calcite, quartz, phosphate (carbonated hydroxyapatite)

Interpretation: fired to 500-600 °C

**Stratum 13**

**Purple Vat 8076/10 (VB)**

This spectrum is characterized by mid-to-low-temperature heated clays, calcite, quartz, phosphate (carbonated hydroxyapatite), and some organic material. The main clay absorbance band is at 1043 cm^-1^, while absorbance bands of structurally bound hydroxyl in clay minerals (in the region from 3600-3400 cm^-1^) are absent, and the band at ~913 cm^-1^ is absent. Following experimental data, this suggests the clay minerals in the ceramic were exposed to approximately 500-600 °C (cf. [2]). The absorbance bands at 1429, 875 and 713 cm^-1^ are indicative of calcite. Quartz is indicated by shoulders at ~1080 and ~1170 cm^-1^, the doublet at 797 and 778 cm^-1^, the band at 695 cm^-1^ and the shoulder at 510 cm^-1^. The presence of carbonated hydroxyapatite is indicated by absorbance bands at 603 and 567 cm^-1^. Organic material, possibly contaminants, are visible in the region from ~3000-2800 cm^-1^.

Mineralogy: Clay (heated, 500-600 °C), calcite, quartz, phosphate (carbonated hydroxyapatite)

Interpretation: fired to 500-600 °C

**Stratum 12**

**Purple Vat 8120**

This spectrum is characterized by mid-to-low-temperature heated clays, calcite, quartz, phosphate (carbonated hydroxyapatite) and organic materials. The main clay absorbance band is at 1040 cm^-1^, while absorbance bands of structurally bound hydroxyls in clay minerals (in the region from 3600-3400 cm^-1^) are absent, and the band at ~915 cm^-1^ is absent. Following experimental data, this suggests the clay minerals in the ceramic were exposed to approximately 500-600 °C (cf. [2]). The absorbance bands at 1426, 875 and 713 cm-1 are indicative of calcite. Quartz is indicated by shoulders at ~1080 and 1171 cm-1, the doublet at 797 and 778 cm^-1^ and the band at 695 cm-1. The presence of carbonated hydroxyapatite is indicated by absorbance bands at 604 and 563 cm^-1^. A shoulder at ~578 cm^-1^ is not confidently identified. Organic material, possibly contaminants, is visible in the region from ~3000-2800 cm^-1^.

Mineralogy: Clay (heated, 500-600 °C), calcite, quartz, phosphate (carbonated hydroxyapatite)

Interpretation: fired to 500-600 °C

**Stratum 11**

**Purple Vat 6313 (PVR)**

This spectrum is characterized by mid-temperature heated clays, calcite, quartz, phosphate (carbonated hydroxyapatite) and organic material. The main clay absorbance band is shifted to 1047 cm^-1^, while absorbance bands of structurally bound hydroxyl in clay minerals (in the region from 3600-3400 cm^-1^) are absent, and the band at ~913 cm^-1^ is absent. Following experimental data, this suggests the clay minerals in the ceramic were exposed to approximately 600 °C (cf. [2]). The absorbance bands at 1427, 875 and 713 cm^-1^ are indicative of calcite. The presence of calcite in this sample gives us an upper limit of ~600-650 °C, as calcite starts to degrade at this temperature [4]. Quartz is indicated by shoulders at ~1080 and ~1170 cm^-1^, the doublet at 797 and 778 cm^-1^, the band at 695 cm^-1^ and the shoulder at 514 cm^-1^. The presence of carbonated hydroxyapatite is indicated by absorbance bands at 604 and 564 cm^-1^. Organic material, possibly contaminants, are visible in the region from ~3000-2800 cm^-1^.

Mineralogy: Clay (heated, ~600 °C), calcite, quartz, phosphate (carbonated hydroxyapatite)

Interpretation: fired to 600 °C

**Purple Vat 6207 (VB)**

This spectrum is characterized by mid-to-low-temperature heated clays, calcite, quartz, phosphate (carbonated hydroxyapatite), and some organic material. The main clay absorbance band is at 1039 cm^-1^, while absorbance bands of structurally bound hydroxyl in clay minerals (in the region from 3600-3400 cm^-1^) are absent, and the band at ~913 cm^-1^ is absent. Following experimental data, this suggests the clay minerals in the ceramic were exposed to approximately 500-600 °C (cf. [2]). The absorbance bands at 1429, 875 and 713 cm^-1^ are indicative of calcite. Quartz is indicated by shoulders at ~1080 and ~1170 cm^-1^, the doublet at 797 and 778 cm^-1^, the band at 695 cm^-1^ and the shoulder at 510 cm^-1^. The presence of carbonated hydroxyapatite is indicated by absorbance bands at 604 and 564 cm^-1^. Organic material, possibly contaminants, is visible in the region from ~3000-2800 cm^-1^.

Mineralogy: Clay (heated, 500-600 °C), calcite, quartz, phosphate (carbonated hydroxyapatite)

Interpretation: fired to 500-600 °C

**Purple Vat 6255/5 (VB)**

This spectrum is characterized by low-heated clays, calcite, quartz, traces of aragonite, phosphate (carbonated hydroxyapatite), and some organic material. The main clay absorbance band is at 1031 cm^-1^, while absorbance bands of structurally bound hydroxyl (OH-) in clay minerals (in the region from 3600-3400 cm^-1^) are absent, and the band at ~913 cm^-1^ is present only as a weak shoulder. Following experimental data, this suggests the clay minerals in the ceramic were exposed to lower temperatures, approximately 400-500 °C (cf. [2]). The absorbance bands at 1429, 875 and 713 cm^-1^ are indicative of calcite. The shoulders at 855 cm^-1^ (and slight shoulders at 1476, 1083, and 700 cm^-1^, more clearly indicated by the first difference second derivative) are indicative of minor traces of aragonite. Quartz is indicated by shoulders at ~1084 and 1171 cm^-1^, the doublet at 797 and 778 cm^-1^ and the band at 695 cm^-1^. The presence of minor amounts of carbonated hydroxyapatite is indicated by small absorbance bands around 605 and 565 cm^-1^. A shoulder at ~578 cm^-1^ is not confidently identified. Organic material, possibly contaminants, are visible in the region from ~3000-2800 cm^-1^. The bands at 2360 and 2340 cm^-1^ are related to carbon dioxide in the air.

Mineralogy: Clay (heated, 400-500 °C), calcite, quartz, aragonite, phosphate (carbonated hydroxyapatite)

Interpretation: fired to 400-500 °C

**Stratum 11 or 8**

**Purple Vat 13A2020 (PVR)**

This spectrum is characterized by mid-to-low-temperature heated clays, calcite, quartz, phosphate (carbonated hydroxyapatite) and organic materials. The main clay absorbance band is at 1039 cm^-1^, while absorbance bands of structurally bound hydroxyl in clay minerals (in the region from 3600-3400 cm^-1^) are absent, and the band at ~915 cm-1 is absent. Following experimental data, this suggests the clay minerals in the ceramic were exposed to approximately 500-600 °C (cf. [2]). The absorbance bands at 1429, 875 and 713 cm^-1^ are indicative of calcite. Quartz is indicated by shoulders at ~1080 and 1170 cm-1, the doublet at 797 and 778 cm^-1^ and the band at 695 cm^-1^. The presence of carbonated hydroxyapatite is indicated by absorbance bands at 604 and 563 cm^-1^. Organic material, possibly contaminants, is visible in the region from ~3000-2800 cm^-1^.

Mineralogy: Clay (heated, 500-600 °C), calcite, quartz, phosphate (carbonated hydroxyapatite)

Interpretation: fired to 500-600 °C

**Stratum 9**

**Purple Vat 7238**

This spectrum is characterized by mid-to-low-temperature heated clays, calcite, quartz, phosphate (carbonated hydroxyapatite) and organic material. The main clay absorbance band is at 1043 cm^-1^, while absorbance bands of structurally bound hydroxyl in clay minerals (in the region from 3600-3400 cm^-1^) are absent, and the band at ~913 cm^-1^ is absent. Following experimental data, this suggests the clay minerals in the ceramic were exposed to approximately 500-600 °C (cf. [2]). The absorbance bands at 1429, 875 and 713 cm^-1^ are indicative of calcite. Quartz is indicated by shoulders at ~1080 and ~1170 cm^-1^, the doublet at 797 and 778 cm^-1^, the band at 695 cm^-1^ and the shoulder at 514 cm^-1^. The presence of carbonated hydroxyapatite is indicated by absorbance bands at 602 and 563 cm^-1^. Organic material, possibly contaminants, are visible in the region from ~3000-2800 cm^-1^.

Mineralogy: Clay (heated, 500-600 °C), calcite, quartz, phosphate (carbonated hydroxyapatite)

Interpretation: fired to 500-600 °C

**Stratum 8**

**Purple Vat 5277 (VB)**

This spectrum is characterized by mid-to-low-temperature heated clays, calcite, quartz, phosphate (carbonated hydroxyapatite), and organic material. The main clay absorbance band is at 1043 cm^-1^, while absorbance bands of structurally bound hydroxyl in clay minerals (in the region from 3600-3400 cm^-1^) are absent, and the band at ~913 cm^-1^ is absent. Following experimental data, this suggests the clay minerals in the ceramic were exposed to approximately 500-600 °C (cf. [2]). The absorbance bands at 1427, 875 and 713 cm^-1^ are indicative of calcite. Quartz is indicated by shoulders at ~1080 and ~1170 cm^-1^, the doublet at 797 and 778 cm^-1^, the band at 695 cm^-1^ and the shoulder at 514 cm^-1^. The presence of carbonated hydroxyapatite is indicated by absorbance bands at 604 and 564 cm^-1^. Organic material, possibly contaminants, is visible in the region from ~3000-2800 cm^-1^.

Mineralogy: Clay (heated, 500-600 °C), calcite, quartz, phosphate (carbonated hydroxyapatite)

Interpretation: fired to 500-600 °C

**Stratum 7**

**Purple Vat 5548 (VB)**

This spectrum is characterized by low-temperature heated clays, calcite, quartz, traces of aragonite, phosphate (carbonated hydroxyapatite), and some organic material. The main clay absorbance band is at 1035 cm^-1^, while absorbance bands of structurally bound hydroxyl in clay minerals (in the region from 3600-3400 cm^-1^) are absent, and the band at ~913 cm^-1^ is present only as a weak shoulder. Following experimental data, this suggests the clay minerals in the ceramic were exposed to lower temperatures, approximately 400-500 °C (cf. [2]). The absorbance bands at 1425, 875 and 712 cm^-1^ are indicative of calcite. The shoulder at 855 cm^-1^ (and slight shoulders at 1476, 1083, and 700 cm-1, more clearly indicated by the first difference second derivative) is indicative of minor traces of aragonite. Quartz is indicated by shoulders at ~1084 and 1171 cm^-1^, the doublet at 797 and 778 cm^-1^ and the band at 695 cm^-1^. Compared to the other spectra, the phosphate peaks are less prominent, although shoulders around 605 and 565 cm^-1^ are indicative of carbonated hydroxyapatite. Organic material, possibly contaminants, is visible in the region from ~3000-2800 cm^-1^.

Mineralogy: Clay (heated), calcite, quartz, aragonite, phosphate (carbonated hydroxyapatite)

Interpretation: fired to 400-500 °C

**Unstratified**

**Purple Vat 6123 (PBVF)**

This spectrum is characterized by mid-to-low-temperature heated clays, calcite, quartz, phosphate (carbonated hydroxyapatite) and organic materials. The main clay absorbance band is at 1039 cm^-1^, while absorbance bands of structurally bound hydroxyl in clay minerals (in the region from 3600-3400 cm^-1^) are absent, and the band at ~913 cm^-1^ is absent. Following experimental data, this suggests the clay minerals in the ceramic were exposed to approximately 500-600 °C (cf. [2]). The absorbance bands at 1428, 874 and 713 cm^-1^ are indicative of calcite. Quartz is indicated by shoulders at ~1080 and ~1170 cm-1, the doublet at 797 and 778 cm^-1^ and the band at 695 cm^-1^. The presence of carbonated hydroxyapatite is indicated by absorbance bands at 604 and 563 cm^-1^. Organic material, possibly contaminants, is visible in the region from ~3000-2800 cm^-1^.

Mineralogy: Clay (heated, 500-600 °C), calcite, quartz, phosphate (carbonated hydroxyapatite)

Interpretation: fired to 500-600 °C

**Commentary**

The sediment at Shiqmona is highly calcareous, in contrast to the clayey colluvial–alluvial vertisols of the nearby Qishon River [7]. This calcareous composition presents a technical limitation for firing ceramic basins, since firing temperatures above 500–600 °C can convert the abundant calcium carbonate into calcium hydroxide (lime), causing the fabric to crumble [5, 8]]. Based on our results, it appears that the basins were fired at the minimum temperature required to transform the clay into pottery. This not only prevented structural failure but likely also helped save fuel during the production of these large basins.

Given that both the ceramic fabric and the dyeing environment were saline, heat—especially if the vats were heated during the production of the dye—would not directly alter the calcium carbonates in the vessels, but it could accelerate the movement and (re)crystallization of salts within the fabric of the ceramic basins [10]. Over time, this process could lead to degradation and micro-fracturing, especially if the basins were subjected to repeated cycles of wetting and drying.

The salt-rich and calcareous environment at Shiqmona suggests that potters and dyers had to carefully prepare, fire and manage vessels over their lifespans, potentially replacing basins as they deteriorated.

1. Butler DH, Shahack-Gross R. Formation of biphasic hydroxylapatite-beta magnesium tricalcium phosphate in heat-treated salmonid vertebrae. Sci Rep. 2017;7(1):3610.
2. Berna F, Behar A, Shahack-Gross R, Berg J, Boaretto E, Gilboa A, et al. Sediments exposed to high temperatures: reconstructing pyrotechnological processes in Late Bronze and Iron Age strata at Tel Dor (Israel). J Archaeol Sci. 2007;34: 358–373. https://doi.org/10.1016/j.jas.2006.05.011.
3. Forget MCL, Regev L, Friesem DE, Shahack-Gross R. Physical and mineralogical properties of experimentally heated chaff-tempered mud bricks: implications for reconstruction of environmental factors influencing the appearance of mud bricks in archaeological conflagration events. J Archaeol Sci Rep. 2015;2:80–93.
4. Shoval S, Paz Y. Analyzing the fired-clay ceramic of EBA Canaanite pottery using FT-IR spectroscopy and LA-ICP-MS. Period Mineral. 2015;84(1):213–31. <https://doi.org/10.2451/2015PM0011>.
5. Waiman-Barak P, Susnow M, Nickelsberg R, Cline EH, Yasur-Landau A, Shahack-Gross R. Technological aspects of Middle Bronze Age II production of pithoi at Tel Kabri, Israel: specialized pottery production in a palatial system. Levant. 2018;50(1):32–51.
6. Vachtman D, Sandler A, Greenbaum N, Herut B. Dynamics of suspended sediment delivery to the Eastern Mediterranean continental shelf. Hydrol Process. 2013;27(7):1105–16.
7. Shoval S. Using FT-IR spectroscopy for study of calcareous ancient ceramics. Opt Mater. 2003;24(1–2):117–22.
8. O'Brien P. An experimental study of the effects of salt erosion on pottery. J Archaeol Sci. 1990;17(4):393–401.
