# Supplementary material for: Tel Shiqmona during the Iron Age: A first glimpse into an ancient Mediterranean purple dye ‘factory’
Source: PLoS One. 2025 Apr 16;20(4):e0321082. doi: 10.1371/journal.pone.0321082 (PMC12002455; doi:10.1371/journal.pone.0321082)
Supplement: S1 Table — Abbreviations: MB=Middle Bronze Age, LB=Late Bronze Age, IR=Iron Age. (DOCX) [file pone.0321082.s003.docx]

**S1 Table:** Sites with direct evidence interpreted as indicating dye manufacturing during the Bronze and Iron Ages around the Mediterranean,* mainly following Reese 2025.** Abbreviations: MB=Middle Bronze Age, LB=Late Bronze Age, IR=Iron Age.

| **Region** | **Site** | **Relative chronology** | **Absolute chronology BCE** | **Evidence** |
| --- | --- | --- | --- | --- |
| Aegean | Alatzomouri-Pefka | MB |  | Dye residue on pottery (N=?) |
| Aegean | Aegina Kolonna | LBIA | 16th century | Two stained body fragments and another six pottery fragments with some residues (N=8; MNI=7?) |
| Cyprus | Pyrgos-Mavroraki | MB? | ? | Few shells and purple/red clots (N=?) |
| Cyprus | Hala Sultan Tekke | IRI | 1175-1110 | Shells and stained basin and soil |
| Cyprus | Kition | IRI | Late 12^th^ to early 11^th^ century | Stained potsherds of one vessel (N=55; MNI=1) |
| Syria | Minet el-Beidha/Ugarit | LB |  | Shells, stained sherd (N=1) |
| Lebanon | Sarepta | LBIIA | 1350–1275 | Stained pottery sherds (N=3; MNI=1) |
|  |  | IRI | 1275-1150 | Stained pottery sherds (N=3; MNI=1) |
| Southern Levant | Tell ‘Akko | LBIIA | 13^th^ to early 12^th^ century | Shells; Stained vessel (N=1) |
| Southern Levant | Tel Shiqmona | IRI–IRIIC | 1100–600 | Stained pottery and stones (N=135) |
| Southern Levant | Tel Dor | IRI, IRIIC |  | Shells, stained pottery sherds (N=3, including a vat fragment) |
| Southern Levant | Tell Keisan | IRI | 11^th^ century | Stained vessel (N=1) |
| Southern Levant | Tel Kabri | IRIIC | 7^th^ century | Stained vessel (N=2, including a vat fragment) |

* Textiles or other finished products alone do not constitute direct evidence for dye production at a site, and therefore not included in the table.

** Reese DS. Shellfish purple colour in East Mediterranean: A Gazetteer of Sites. In: Mylona D, Brogan TM, Eaby M, Iacovou M, editors. PORPHYRA: the materiality of purple dye production and use in Cyprus and the Aegean from prehistory to the Late Roman period. Louvain: Aegis; forthcoming 2025. p. 147–194.
