# Supplementary material for: Tel Shiqmona during the Iron Age: A first glimpse into an ancient Mediterranean purple dye ‘factory’
Source: PLoS One. 2025 Apr 16;20(4):e0321082. doi: 10.1371/journal.pone.0321082 (PMC12002455; doi:10.1371/journal.pone.0321082)
Supplement: S2 Table — (DOCX) [file pone.0321082.s004.docx]

**S2 Table:** The Iron Age stratigraphic/chronological sequence at Tel Shiqmona

| **Stratum** | **Main Characteristics** | **Period** | **Date BCE** | **Material culture** |
| --- | --- | --- | --- | --- |
| 15b | Village, purple dye production, destroyed | Iron Age IB | 11^th^ century | Local Carmel Coast (‘Phoenician’) |
| 15a | Village, purple dye production, abandoned | Iron Age IB | 11^th^–first half of the 10^th^ century | Local Carmel Coast (‘Phoenician’) |
| 14 | Village, purple dye production, destroyed? | Early Iron Age IIA | Second half of the 10^th^ century | Local Carmel Coast (‘Phoenician’) |
| 13 | Casemate enclosure, purple dye industry, violently destroyed | Late Iron Age IIA | 9^th^ century | Mixed inland (Israelite) and coastal (‘Phoenician’) |
| 12 | Casemate enclosure, purple dye industry, oil production | Late Iron Age IIA | ~830/820–790 | Mixed inland (Israelite) and coastal (‘Phoenician’) |
| 11 | Casemates only partly used, extramural settlement extension, purple dye industry, oil production, violently destroyed | Iron Age IIB | ~790-740 | Mixed inland (Israelite) and coastal (‘Phoenician’) |
| 10 | Large Four Room House with olive oil press, purple dye production, destroyed | Iron Age IIB | ~740–700 | Mixed inland (Israelite) and coastal (‘Phoenician’) |
| 9 | Poor architecture, renewal of purple dye production | Iron Age IIC | ~700–675 | Mixed inland (Israelite) and coastal (‘Phoenician’) |
| 8 | Purple dye and olive oil industrial quarter under Assyrian domination | Iron Age IIC | ~675–645 | Coastal (‘Phoenician’) |
| 7 | Purple dye industrial quarter | Iron Age IIC | ~645–600 | Coastal (‘Phoenician’) |
