# Supplementary material for: Tel Shiqmona during the Iron Age: A first glimpse into an ancient Mediterranean purple dye ‘factory’
Source: PLoS One. 2025 Apr 16;20(4):e0321082. doi: 10.1371/journal.pone.0321082 (PMC12002455; doi:10.1371/journal.pone.0321082)
Supplement: S4 Table — Abbreviations: PVR = purple-stained vat rim, PVBF = body fragment of vat with purple remains, PSH = purple-stained miscellaneous potsherd, PST = purple-stained stone tools and object. Items marked with an asterisk are from Bar’s excavation and were published in Sukenik et al. 2017. (DOCX) [file pone.0321082.s006.docx]

**S4 Table:** Results of the HPLC; the ratios of the identified components at 554. Abbreviations: PVR= purple-stained vat rim, PVBF= vat body fragment with purple remains. Items marked with an asterisk (*) are from the Bar excavations at Shiqmona and were previously published by Sukenik *et al.* (2017).

| **Site/no sample** | **Material** | **Type** | **Locus/basket** | **IND** | **MBI** | **MBIR** | **DBI** | **DBIR** |
| --- | --- | --- | --- | --- | --- | --- | --- | --- |
| Shiqmona 1 | Ceramic | PVBF | 2091/13023 | 14.4% | 41.8% | 0% | 36.3% | 7.6 |
| Shiqmona 2 | Ceramic | PVBF | 13A-286/2193 | 29.05% | 40.26% | 2.62% | 16.65% | 11.42% |
| Shiqmona 3 | Ceramic | PVBF | 265/8053 | 22.80% | 48.14% | 0.90 | 21.37% | 6.79% |
| Shiqmona 4 | Ceramic | PVBF | 12B-713/7012 | 34.82% | 42.12% | 2.11% | 12.18% | 8.75% |
| Shiqmona 5* | Ceramic | PVBF | 11B-224/2051-1 | 34% | 47.3 | 2.5% | 5.7% | 10.1% |
| Shiqmona 6 | Ceramic | PVBF | 11B-224/2051-2 | 36.7% | 41.5% | 3.71% | 4.42% | 12.56% |
| Shiqmona 7 | Ceramic | PVR | 13A-206/2020 | 19% | 48.01% | 1.1% | 12% | 19.22% |
| Shiqmona 8 | Ceramic | PVR | 12B-701/7005 | 20.07% | 34.8 | 2.33% | 22.15% | 19.91% |
| Shiqmona 9* | Ceramic | PVBF | 11B-243/2077-1 | 31.38 % | 46.25 % | 2.40 % | 10.44% | 9.53% |
| Shiqmona 10* | Ceramic | PVBF | 11B-239/2068 | 35.1% | 41% | 2.5% | 11.4% | 10% |
| Shiqmona 11 | Ceramic | PVBF | 472/7238 | 33.2% | 34.5% | 2.2% | 12.3% | 16.6% |
| Shiqmona 12 | Ceramic | PVBF | 2278/5336 | 33.2% | 34% | 2.3% | 22.4% | 7.7% |
| Shiqmona 13 | Ceramic | PVBF | Installation 2/5277 | 26.20% | 27.33% | 3.69% | 9.86% | 32.88% |
| Shiqmona 14* | Ceramic | PVBF | 11B-329/2299 | 41.1% | 34.3% | 0.6% | 16.2% | 7.9% |
| Shiqmona 16 | Ceramic | PVBF | 11B-243/2077-2 | 31.4% | 48% | Under 0.1% | 14.3% | 6.2 |
| Shiqmona 17 | Ceramic | PVBF | 665/7190 | 39.18% | 26.73% | 3.67% | 9.08% | 21.32% |
| Shiqmona 18 | Ceramic | PVBF | 274/7111 | 34.5% | 41.1% | 0.7% | 16 % | 7.7% |
| Shiqmona 19 | Ceramic | PVBF | 2261/6269 | 14.3% | 43.6% | 0.8% | 20.6% | 20.7% |
| Shiqmona 20 | Ceramic | PVR | 274/6296 | 30.6% | 32.4% | 4.2% | 9.6% | 21.3 |
| Shiqmona 21 | Ceramic | PVR | 2262/6313 | 29% | 44.6% | 1.7% | 17.5% | 7.2 |
